# Multiple introductions of equine influenza virus into the United Kingdom resulted in widespread outbreaks and lineage replacement

**DOI:** 10.1371/journal.ppat.1013227

**Published:** 2025-06-09

**Authors:** Laura Mojsiejczuk, Fleur Whitlock, Hanting Chen, Callum Magill, Elihu Aranday-Cortes, Jordan Bone, Lily Tong, Ana Da Silva Filipe, Neil Bryant, J. Richard Newton, Thomas M. Chambers, Stephanie E. Reedy, Manabu Nemoto, Takashi Yamanaka, Joseph Hughes, Pablo R. Murcia

**Affiliations:** 1 MRC-University of Glasgow Centre for Virus Research, Glasgow, United Kingdom; 2 Department of Veterinary Medicine, University of Cambridge, Cambridge, United Kingdom; 3 Department of Veterinary Science, Maxwell H. Gluck Equine Research Center, University of Kentucky, Lexington, Kentucky, United States of America; 4 Equine Research Institute, Japan Racing Association, Shimotsuke, Japan; University of North Carolina at Chapel Hill, UNITED STATES OF AMERICA

## Abstract

Influenza A viruses (IAVs) are prime examples of emerging viruses in humans and animals. IAV circulation in domestic animals poses a pandemic risk as it provides new opportunities for zoonotic infections. The recent emergence of H5N1 IAV in cows and subsequent spread over multiple states within the USA, together with reports of spillover infections in humans, cats and mice highlight this issue. The horse is a domestic animal in which an avian-origin IAV lineage has been circulating for >60 years. In 2018/19, a Florida Clade 1 (FC1) virus triggered one of the largest epizootics recorded in the UK, which led to the replacement of the Equine Influenza Virus (EIV) Florida Clade 2 (FC2) lineage that had been circulating in the country since 2003. We integrated geographical, epidemiological, and virus genetic data to determine the virological and ecological factors leading to this epizootic. By combining newly-sequenced EIV complete genomes derived from UK outbreaks with existing genomic and epidemiological information, we reconstructed the nationwide viral spread and analysed the global evolution of EIV. We show that there was a single EIV FC1 introduction from the USA into Europe, and multiple independent virus introductions from Europe to the UK. At the UK level, three English regions (East, West Midlands, and North-West) were the main sources of virus during the epizootic, and the number of affected premises together with the number of horses in the local area were found as key predictors of viral spread within the country. At the global level, phylogeographic analysis evidenced a *source-sink* model for intercontinental EIV migration, with a source population evolving in the USA and directly or indirectly seeding viral lineages into sink populations in other continents. Our results provide insight on the underlying factors that influence IAV spread in domestic animals.

## Author summary

Influenza causes significant disease burden in animals, including wild birds, sea lions, pigs, horses, dogs, and more recently, cows. Outbreaks and epizootics of influenza in agricultural species are a threat to food security and the economy whereas in wild animals they could affect biodiversity and conservation efforts. Given the zoonotic nature of influenza viruses and the high levels of contact between domestic animals and humans, animal influenza is also a public health concern. Here, we combined geographical, epidemiological, and virus sequence data to determine key factors that led to one of the largest epizootics of equine influenza in the United Kingdom in decades. We show that an American equine influenza virus lineage was introduced into Europe and replaced the virus lineage that had been circulating in the United Kingdom for nearly 20 years. We also analysed a global dataset of virus genomes and propose a model of equine influenza virus intercontinental migration, in which USA is the main source of viruses to other countries. Our results provide important information concerning the basic principles of influenza virus circulation in animal populations. This is central to devise effective measures of disease control that would increase animal health while reducing zoonotic risk.

## Introduction

Host genetics, immune competence, population structure and spatial ecology are some of the various factors inextricably linked to the evolution and epidemiology (i.e., phylodynamics) of pathogens [1]. Therefore, viral phylogenies can be used to assess fundamental biological processes, such as epidemic spread, zoonotic transmission, antigenic drift and selective sweeps [2]. In recent years, phylodynamic methods have contributed significantly to our understanding of infectious disease dynamics. For example, studies linking pathogen evolution, epidemiological information, and host movement data provided insights into the source and geographical distribution of human influenza, while identifying air travel as a key underlying factor that drives its global spread [3]. They also helped to track the early spread of SARS-CoV-2, overcoming the lack of genomic information from some affected countries [4]; and identified the impact of agricultural land use on West Nile virus emergence and spread in Europe [5]. A practical benefit of phylodynamic approaches is that they can reveal hidden transmission patterns that are inaccessible via traditional epidemiological analyses. For example, Wohl *et al.*combined pathogen genomics with epidemiological data and patient information to link two seemingly unrelated mumps outbreaks that affected different communities in Massachusetts, USA [6], whereas Müller *et al.* used a similar approach to reveal distinct influenza transmission networks in children and the elderly in Basel, Switzerland [7]. Identifying drivers that impact the spatiotemporal incidence of infections at regional scales is essential to develop effective measures to reduce disease burden, such as targeted vaccination of children to control influenza [8].

Influenza A viruses (IAVs) cause significant disease burden in various mammalian and avian species [9]. While birds are considered the main reservoir of IAVs, they have crossed into and established endemic lineages in humans, dogs, pigs and horses [9,10]. IAV phylogenetic patterns vary according to host species or even viral subtype: human H3N2 IAV exhibits a single global lineage with high strain turnover, whereas human H1N1 shows less frequent selective sweeps with multiple lineages persisting across seasons [11]. Animal strains, like swine and equine IAVs, are frequently characterised by the co-circulation of geographically segregated lineages [12,13]. The size and connectivity of host populations play an important role in the maintenance and dynamics of IAV lineages [14–16]. As a result, globalisation and changes in population structure (e.g., intensive farming) can lead to increased competition between viral lineages, changes in selective pressures, increased diversification, and pathogenicity [17]. Recently, the highly pathogenic avian H5N1 strain has emerged in cattle in the USA and spread over multiple states [18]. Further, H5N1 IAV spillover infections have been reported in cats and mice [18,19]. Zoonotic infections in humans have also been reported, but the source of virus is unclear [20].

Equine influenza (EI) is a highly transmissible disease of equids. Outbreaks and epizootics of EI can have a significant socio-economic impact in communities that rely on working equids [21,22], as well as in countries where horse competitions attract international travel attendance [23]. There is currently only one equine influenza virus (EIV) subtype (H3N8), which has been circulating continuously since 1963 when it was first detected [24]. EIV vaccines have been available for decades, but their use varies according to national regulations. For example, vaccination is mandatory for competition horses in some countries such as the UK [25]. Mandatory requirements for vaccination of racehorses were introduced in Europe in 1981 after major disruptions to racing due to EI occurred in 1979 [26]. Antigenic drift is common for EIV, and the World Organisation for Animal Health (WOAH, formerly OIE) hosts an annual meeting of an expert surveillance panel on EI vaccine composition that recommends which virus strains should be included in vaccines [27]. The long-term phylogeny of EIV has been described previously [23 ] showing that diverging lineages coexist (such as Florida Clade 1 [FC1] and Florida Clade 2 [FC2] in America and Europe between 2003 and 2019), consistent with endemic EIV circulation in geographically isolated equine populations on different continents [12,28]. However, the detection of foreign lineages is not unusual and is frequently associated with recently imported animals, including those involved in the international circuit of sales, breeding, and equestrian sports events. While these introductions may be detected during quarantine procedures, which can facilitate successful containment, such protocols are not always implemented. There can be spread to local horse populations, or, as happened in Australia in 2007 (where the resident population, due to the complete absence of disease nationally, was not required to be vaccinated) they can trigger nationwide epizootics [29]. A key unanswered question is: which factors facilitate epidemic burn-out and which enable continued spread? While antigenic differences between the infecting and immunising strains affect the risk and size of influenza outbreaks [30,31], other factors such as host population structure, population level protective immunity, and the quality and extent of biosecurity measures implemented at local and national level, including those affecting host movements, are likely to play an important role in the outcome of viral introductions [32].

The UK has a dedicated surveillance scheme for equine infectious diseases funded by the Horserace Betting Levy Board (HBLB) [33]. This scheme assists veterinary surgeons in confirming influenza cases while also encouraging testing in vaccinated horses presenting with non-specific respiratory signs. Epidemiological and sequencing data are gathered to determine the effectiveness of current vaccines. Between December 2018 and September 2019, one of the largest EI epizootics was recorded in Great Britain (GB) (hereafter referred to as FC1 EI epizootic), with over 200 outbreaks reported, with a total of 412 horses from 234 premises distributed among 65 of the 100 GB counties confirmed as EIV-positive by qPCR [34]. Intervention measures, including vaccination and temporal mobility restrictions, were put in place during the initial phases of the epizootic [34]. The high surveillance coverage during the epizootic provided epidemiological data and virus samples for genomic sequencing to study viral, host, and spatial factors driving influenza epizootic dynamics with an unprecedented level of detail.

In this study, we generated complete genome sequences for more than 50% of the FC1 EI outbreaks in the UK. We combined genomic data with multiple sources of epidemiological information collected during the epizootic, and with global EIV genomic data available in publicly accessible repositories. Using phylodynamic approaches we reconstructed the spatiotemporal spread of EIV within the UK and proposed a model of EIV intercontinental circulation.

## Results

### The 2018/19 EI epizootic in Europe started with a viral introduction of a single reassortant virus from North America

**Fig 1:**
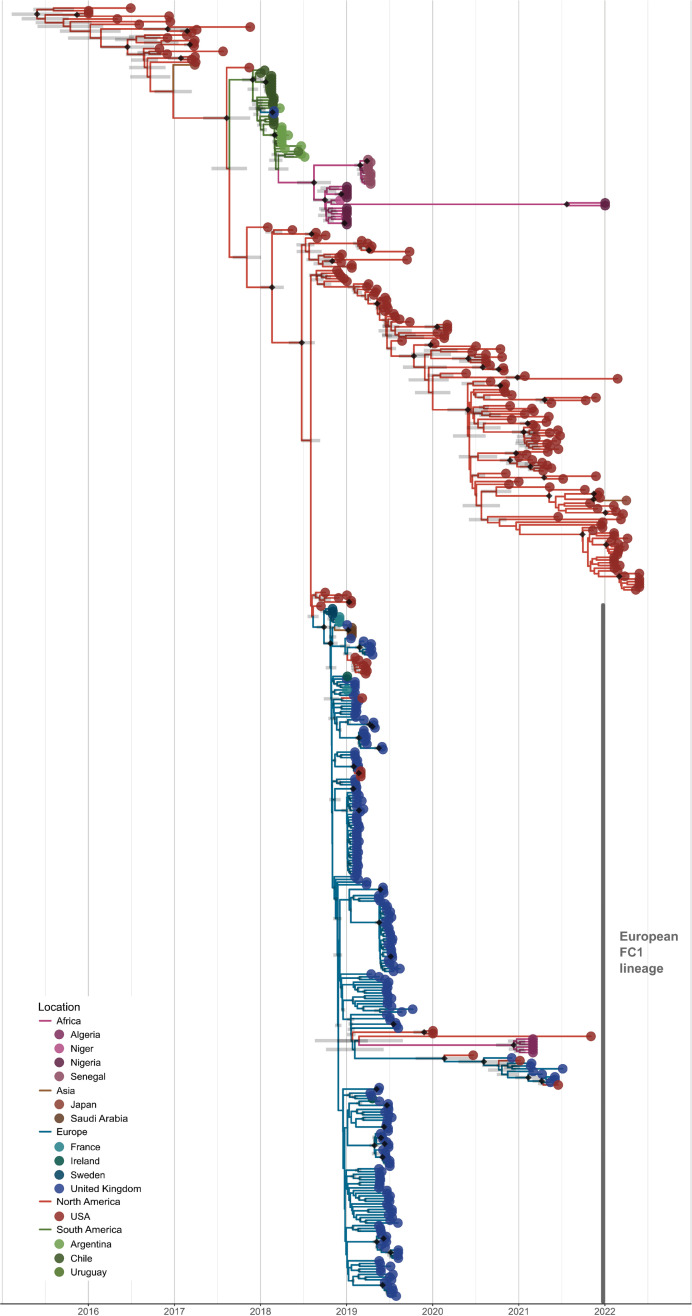
Time-resolved phylogeny from the discrete phylogeographic analysis of Florida Clade 1. MCC tree obtained from the analysis of the HA-only dataset. Branches are coloured according to the most probable location of the parental node of each lineage (colour codes are shown in the lower left) across the five defined global regions. Tips representing European sequences are coloured based on their country of origin. Diamonds on nodes represent posterior probability support ≥ 0.9, with grey horizontal bars representing the 95% HPD estimates for node age.

The first reported outbreak of FC1 EI in Europe was from France on December 14th 2018, and the first detection of FC1 EIV in the UK was on December 28th 2018 [34,35]. To identify the viral source of the European epizootic and when it started, we examined two datasets (see methods): an haemagglutinin (HA)-only dataset (n = 319) and a complete genome dataset (n = 204), that included all FC1 sequences available in public databases, the sequences of the UK 2019 epizootic, and 33 newly generated complete EIV genomes from the USA. Fig 1 shows that the FC1 lineage associated with the European epizootics has a single genetic origin with an ancestor in North America, suggesting a single introduction from that continent into Europe. The common genetic origin is also observed in the phylogenies obtained from the complete genome dataset (S1–S8 Figs). Notably, detailed analysis of the maximum likelihood (ML) phylogenies indicates that the FC1 EIV that was introduced to Europe was a reassortant virus that included genomic segments 1, 2, 3, 6 and 7 derived from viruses like A/equine/Idaho/1/2018 and segments 4 and 5 from viruses like A/equine/Washington/2/2018 (S9 Fig). The earliest FC1 EIV sequences from Europe were derived from horses sampled in Sweden on November 1st, 2018. The most recent common ancestor (MRCA) of the European viruses was dated in early October 2018 (median 2018-10-05, HPD95 2018-08-27 to 2018-10-29) suggesting that FC1 EIV had been circulating in Europe approximately nine weeks before the first EI outbreak was reported in France. All European viruses detected since December 2018 displayed the amino acid substitution Ala387Thr in HA. Beyond that, all the early viruses were identical or showed unique substitutions in this genomic segment (see ML tree in S10 Fig). Of note, some EIV sequences dated in March 2019 and collected in California clustered among UK sequences in the phylogenetic tree, suggesting backwards viral movement from either the UK or Europe into the USA. After September 2019, the number of reported EI cases in the UK fell to endemic levels [36]. Notably, the FC2 lineage was replaced by FC1 EIV, with no cases of FC2 EIV confirmed in the UK since last being detected in October 2017. Surveillance reports by the WOAH suggest that such lineage replacement also took place in Europe [27]. Moreover, phylogenetic analysis of available HA sequences derived from UK cases diagnosed in 2020 and 2021 and those collected in the USA in 2019–2021 shows patterns of divergent evolution consistent with a continuous and largely independent circulation of FC1 EIV in the UK (and likely in Europe) and North America (Fig 1).

**Fig 2:**
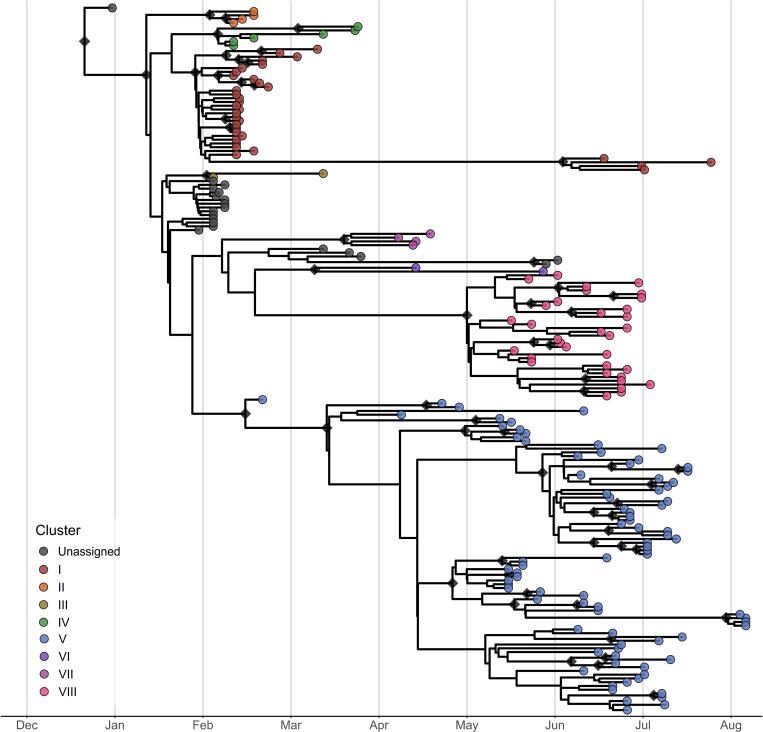
Time-resolved phylogeny of EIV viruses from the UK. The tips in the maximum clade credibility tree are coloured according to the viral clusters, while singletons and low-support clusters are shown in grey. Nodes with posterior probability ≥ 0.9 are labelled with a diamond.

To focus on the epizootic dynamics at the national level, we restricted our analyses to sequences obtained only in the UK. First, we identified the number of introductions and the number of successful transmission clusters within the country. A total of eight viral clusters were observed (Figs 2 and S11).

In this context, a viral cluster was defined as a monophyletic group of two or more sequences sampled from different premises, with posterior probability ≥0.9 in the Bayesian MCC tree, meant to capture viruses that shared a common ancestor and likely originated from the same viral source. Twenty sequences were not assigned to any cluster and exhibited limited or no transmission (referred to as singletons). Due to the low number of background sequences from other countries, and the low genetic diversity accrued in the complete genomes, genomic data alone could not definitively determine if a viral cluster came from a single introduction into the UK, multiple introductions aggregated or cryptic circulation within the UK. However, for clusters I, IV and V, available epidemiological information is consistent with external introductions from Ireland (S1 Table). Therefore, the eight inferred clusters could represent an underestimate of the total number of introductions, increasing further if singletons are accounted as independent viral entries.

The estimated date of the MRCA for each viral cluster is shown in Table 1. All inferred MRCA dates were after the first EI case reported in the UK (December 28th, 2018). The FC1 EIV epizootic displayed two distinct phases: the first phase spanned from late December to March 31st and reached its peak in February. The second phase extended from May 1st to August 31st, with cases peaking in mid-June [34]. During the first phase most detected viruses were associated with Cluster I (13 of 28 affected premises with sequences available), showing evidence of sustained local transmission of this lineage. Singleton viruses, as well as viruses assigned to Clusters II, III, IV and V, affected few premises for short periods, consistent with multiple independent introductions during this phase. The interphase period (April 2019) was characterised by fewer detections of viruses associated with Clusters V, VI, and VII. The subsequent second phase of the epizootic was dominated by local transmissions as an expansion of Cluster V viruses was observed together with the appearance of viruses associated with Cluster VIII. Unassigned viruses, as well as those associated with Clusters I and VI, were detected sporadically. Overall, the period between the estimated MRCA date and the first sequence from each cluster ranged from 2 to 33 days, suggesting that some viruses were in circulation for weeks before being sampled, even in the context of heightened awareness and testing as occurred in the UK horse population in 2019 (Table 1). The time between the first and last detection of a virus from a given cluster ranged from 1 to 23 weeks, with the latter observed for Clusters I and V, which were detected in both phases of the epizootic. Finally, co-circulation of clusters was detected in three premises during the first phase, involving viruses from the following clusters: III/IV/unassigned; III/unassigned; and I/V. In two of these facilities, the confirmed cases were horses imported from Ireland that showed clinical signs of EI on arrival. Evidence of inter-cluster reassortment was investigated, and none of the methods evaluated were able to detect signals of such events. Even after visually inspecting and comparing the trees of individual segments, it was not possible to discriminate between reassortment events and homoplasy due to the low number of signature substitutions for each cluster (ranging from zero to five per genomic segment).

**Table 1:** Characteristics of the viral clusters detected in the UK during the 2019 epizootic.

Cluster	Number of affected premises	MRCA date (median)	MRCA date (HPD95)	Detection lag (days)	Contention lag (days)	Posterior probability
I	17	2019-01-30	[2019-01-19 - 2019-02-06]	12	165	0.91
II	3	2019-02-03	[2019-01-24 - 2019-02-10]	8	7	1
III	2	2019-02-02	[2019-01-29 - 2019-02-03]	2	38	0.92
IV	4	2019-02-07	[2019-01-30 - 2019-02-10]	4	43	0.92
V	66	2019-02-12	[2019-02-07 - 2019-02-20]	9	167	0.99
VI	2	2019-03-16	[2019-02-17 - 2019-04-04]	30	44	0.93
VII	4	2019-03-22	[2019-03-05 - 2019-04-03]	18	11	1
VIII	22	2019-05-03	[2019-04-19 - 2019-05-13]	15	48	1

The most recent common ancestor (MRCA) date (median) was obtained from the BEAST analysis. Detection lag: time between the estimated MRCA date and the first sequence; contention lag: time between the first and last sequence from a cluster; posterior probability: support value for the group in the MCC tree.

### EIV geographical spread is consistent with a complex migration pattern and a highly connected host network

**Fig 3:**
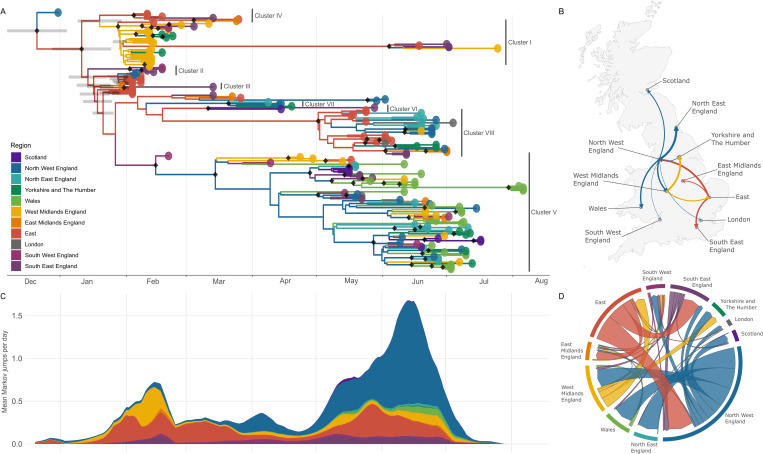
Phylodynamics of viruses isolates during the FC1 EI epizootic in the United Kingdom. **(A)** Time-resolved phylogeny of UK viruses with the summary of the phylogeographic ancestral state reconstruction. Colours on tips and branches correspond to the regions outlined in the upper-right inset. Nodes with posterior probability ≥ 0.9 are labelled with diamonds. **(B)** Supported lineage dispersal events between locations. The thickness of the arrows indicates the corresponding standard BF; only transitions with strong support BF ≥ 20 are plotted. Base UK map shapefile sourced from Natural Earth (https://www.naturalearthdata.com/) public domain. **(C)** Contribution of each region in seeding viral lineages to (any) other locations through time measured as the mean count of Markov jumps per day across all trees in the posterior distribution and smoothed using a 7-day centred rolling mean. **(D)** Between-region circular migration flow plot as estimated from the Markov jumps analysis. Arrows indicate the direction of the migration and thickness is relative to the number of jumps. Only migration events associated with a BF support ≥3 are reported.

To trace the patterns of EIV dispersal in the UK during the 2019 epizootic we performed phylogeographic ancestral state reconstruction using BEAST (see methods). Fig 3A shows that viruses collected from different regions are intermixed in the phylogeny and only a few monophyletic clusters from the same geographical region are present. This suggests either a complex viral migration pattern or a highly connected network (or a combination of these). Additional analysis using BaTS showed no association between geographical location and phylogenetic clustering, the only exception being Wales, where evidence of regional viral clusters was found (S2 Table). We further summarised supported regional transitions in the BEAST phylogeny using the Bayes Factor test (BF). This analysis shows positive evidence (BF ≥ 3) for virus migrations between both close and distant areas (S3 Table). When locations were filtered by high significance values (BF ≥ 20, strong evidence), we observed that East, West Midlands, and North-West regions of England were the most relevant sources of EIV to other UK locations (Fig 3B). Since the level of statistical support for a particular migration link does not inform the relative importance of that transition or migratory route, we estimated the number of transitions or migration events between locations using a Markov jump count procedure in BEAST. This analysis showed that the three English regions: East, West Midlands, and North-West were the major sources of viruses to other regions of the UK, as they constituted the origin of >70% of the migration events during the epizootic showing positive evidence according to the BF test (Fig 3D). The North-West of England was the region with the highest cumulative number of outward migrations. This result, together with well-supported connections (BF ≥ 3) to all locations except the East of England, underscored the relevance of the North-West of England as an epicentre of EIV spread during the FC1 EI epizootic. Further, the geographical source of viruses changed as the epizootic progressed: the East of England and the West Midlands of England were the predominant sources of viral strains during the first peak (February), while the North-West of England was an important source of viruses from late March, becoming dominant in the second phase (Figs 3C and S12). Also, the direction of virus movement changed during different epizootic phases (S13 Fig). For example, the West Midlands acted as a virus source during the first peak but as a receiver during the second wave. During the second phase, the North West was a major source of virus to other regions while also importing virus from different areas. All the other regions can be identified as populations mainly receiving viruses and having low viral export throughout the entire epizootic. The only region that served as a source of virus consistently during the whole epizootic was the East of England. Finally, we complemented the analysis with the obtention of the Markov rewards, a value representing the time a lineage spent in a given region between two migration events and as a measure of the time during which viruses evolve locally [37]. The East and the North West of England have the highest reward time, followed by the West Midlands of England and Wales, indicating longer local viral circulation in these regions (S14 Fig).

To evaluate the impact of the sampling bias across UK regions and its influence on phylogeographic inferences, we conducted additional analyses using downsampled datasets. The North-West and East of England remained as a primary source regions (S15A Fig). However, some differences emerged compared to the original analysis. When sequences were balanced by the number of confirmed cases, the patterns were largely consistent with the original dataset, except for a reduced proportion of migrations from the North-West of England. Balancing sequences by the number of outbreaks led to a more pronounced decrease in Markov jump counts from the North-West, while the West Midlands showed an increased contribution, becoming the second most relevant source region (after the East of England). As expected, when the number of sequences per region was extremely reduced to three, matching the number of sequenced cases in London, the results showed a high variability of patterns across the different regions reflecting the influence of the randomly selected sequences (S15B Fig).

We applied a hierarchical phylogenetic model (HPM) to further investigate which transitions dominate viral spread within the UK when only the supported viral clusters are considered. The joint estimation of the transition matrix across the separate viral clusters largely agrees with the transitions identified in the full dataset analysis (i.e., all UK sequences sharing a single phylogeny), especially for those transitions with strong support (BF ≥ 20; see S3 Table). In contrast, transitions with positive but not strong support (3 ≤ BF < 20) showed less consistency between the two approaches. Overall, this hierarchical analysis reinforces the major phylogeographic patterns inferred from the full dataset, while providing a more conservative view that avoids artefactual transitions across low-support nodes and inferences for the basal branches connecting clusters.

To identify the drivers of virus spread across the UK we performed a phylogeography-based GLM analysis. The only predictors associated with the frequency of virus migration (i.e., BF support ≥3) were the number of EI-affected premises, the number of horses in the premises area at the origin and shared borders (S16 Fig and S4 Table). Surprisingly, the travel distance between regions were not correlated with viral spread. The proportion of sequenced cases in a region was not correlated with virus migration, suggesting that the impact of sampling bias is limited in this analysis.

### Vaccines did not provide sterilising immunity, but reduced viral loads

**Fig 4:**
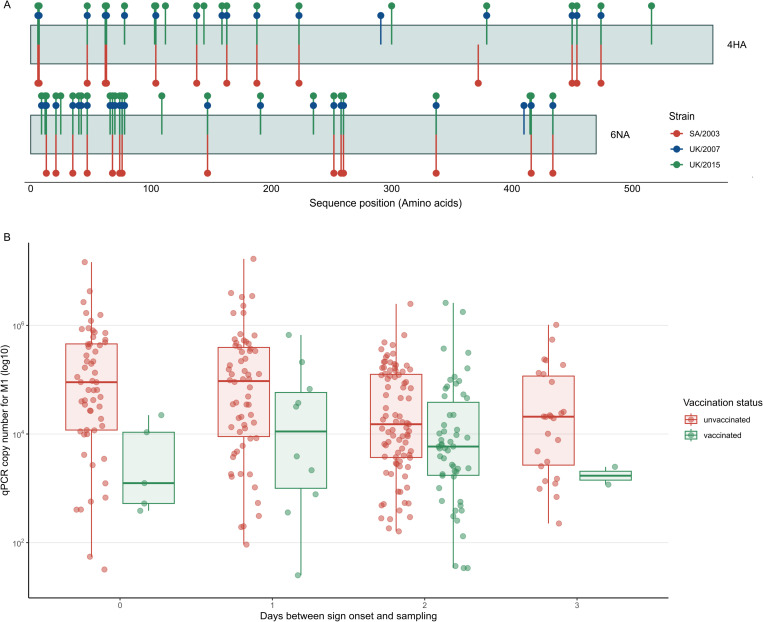
Comparison of EIV HA and NA protein sequences. **(A)** Amino acid changes on surface glycoproteins between A/equine/Lincolnshire/00620/2019 (UK/2019) and the vaccine strains commonly used: A/equine/South Africa/4/2003 (SA/2003) and A/equine/Richmond/1/2007 (UK/2007), and a more recent FC2 strain A/equine/Kent/2015 (UK/2015). **(B)** Viral shedding in vaccinated and unvaccinated horses affected during the 2019 epizootic in the UK. Viral load (determined by qPCR) in vaccinated (n = 74) and unvaccinated horses (n = 297) stratified by day since the onset of clinical signs.

The FC1 EI epizootic was the largest in the UK in decades and affected vaccinated horses. From an immunological standpoint, this is noteworthy because vaccination is mandatory for some horses attending certain forms of competition, such as Thoroughbred racehorses. Since antigenic changes between immunising and infecting IAV strains affect the probability of an influenza epidemic [30,37], we examined the number of amino acid changes at antigenic sites in HA between A/equine/Lincolnshire/00620/2019 (UK/2019) and the two WOAH recommended vaccine strains: A/equine/South Africa/4/2003 (SA/2003) and A/equine/Richmond/1/2007 (UK/2007), as the FC1 lineage and FC2 lineage representative viruses, respectively. A total of 16 amino acid (aa) changes were observed between UK/2019 and SA/2003 (5 of which were at antigenic sites), and 22 aa changes between UK/2019 and UK/2007 (7 mutations at antigenic sites). When we compared the protein sequences of neuraminidase (NA, the other major viral glycoprotein), there were 14 and 24 amino acid changes between UK/2019 and SA/2003 and UK/2007, respectively. We also examined changes in HA and NA between UK/2019 and A/equine/Kent/2015 (UK/2015), the last FC2 EIV that was fully sequenced from a UK outbreak before the FC1 EI epizootic. This comparison showed 26 amino acid mutations in HA (8 of which were at antigenic sites) and another 26 amino acid changes in NA (Fig 4A and S5 Table). To assess if prior EI vaccination led to reduced viral loads in infected horses, we examined qPCR data from diagnostic test results collected from infected horses during the epizootic. Fig 4B shows higher viral loads in the non-vaccinated group. These differences were statistically significant (S6 Table), suggesting that even though vaccines did not provide sterilising immunity, they reduced virus shedding.

### The global migration dynamics of EIV

**Fig 5:**
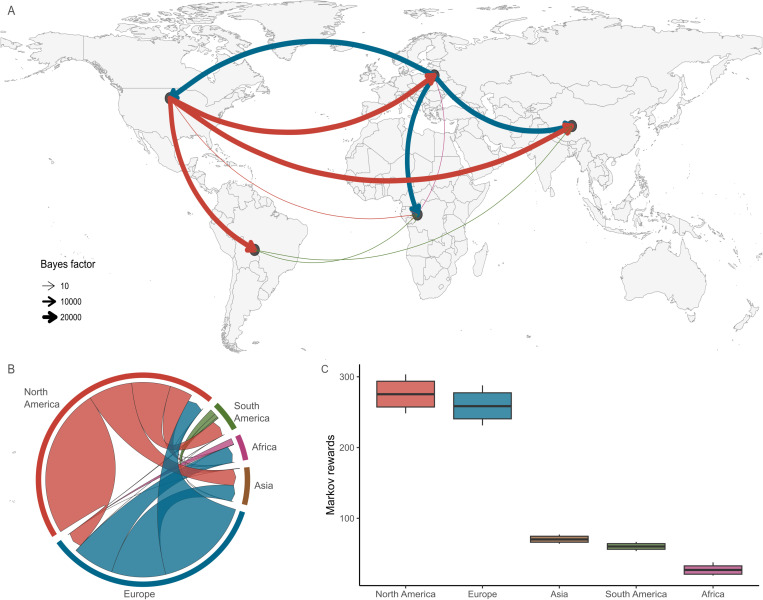
Phylodynamics of the H3N8 EIV lineage between 1963 and 2022. **(A)** Supported lineage dispersal events between locations. The thickness of the arrows indicates the corresponding standard BF; only transitions with positive support BF ≥ 3 are plotted. Base map shapefile sourced from Natural Earth (https://www.naturalearthdata.com/) public domain. **(B)** Between-region circular migration flow plot as estimated from the Markov jumps analysis. Arrows indicate the direction of the migration and thickness is relative to the number of jumps. Only migration events associated with a BF support ≥3 are reported. **(C)** Markov reward times per region. The boxplot of each region depicts the density distribution of the total time (boxes show the median and HPD80 interval).

To identify the international transmission patterns of EIV, we conducted a phylodynamic analysis using 698 HA sequences dating back to the initial detection of H3N8 EIV in 1963. The map in Fig 5A illustrates the supported transitions (BF > 3) that describe the spread of H3N8 EIV throughout its evolutionary history. Our analysis shows the most relevant transitions that explain the international spread of EIV: viruses from North America have migrated to Africa, Asia, Europe and South America, whereas viruses from Europe have migrated to Africa, Asia and North America, but not to South America. Additionally, virus migration from South America to Asia was detected. The number of migration events between regions estimated from the Markov jumps analysis shows that North America is quantitatively the primary source of viral export, accounting as the origin for ~55% of the migration events, followed by Europe at 41.5% (Figs 5B and S17). Africa, Asia and South America made only minimal contributions as sources to the international spread of EIV, acting primarily as viral sinks. Interestingly, Europe is the main receiver of viruses, with North America being the almost exclusive source, and South America and Africa being associated with specific introduction events. The temporal distribution of migration events indicates that the roles of North America and Europe as viral sources for the international circulation of EIV have remained consistent since the 1960s (S18 Fig). However, while North America has remained a constant source with fluctuations over the years, Europe has experienced peaks at specific time points, with the major one occurring during the 2019 epizootic. Lastly, the Markov rewards show that North America and Europe have the highest rewards, consistent with endemic evolution, while the remaining continents display lower values, consistent with viral introductions followed by a short period of transmission, finally leading to the extinction of the lineage (Fig 5C).

To assess the impact of sampling biases, we analysed a downsampled dataset as described in methods. The results from this validation closely mirrored the patterns observed in the original analysis, highlighting the importance of North America and Europe in i) the endemic evolution of EIV and ii) the spread of EIV lineages to other regions (S19 Fig).

## Discussion

The dynamics of infectious diseases are defined by the interplay between ecological, epidemiological, evolutionary and immunological factors. Understanding the processes that drive the epizootic spread of pathogens in domestic animals is an economic priority for infectious diseases that affect food production such as foot and mouth disease, and a public health priority for zoonotic diseases such as rabies or influenza. Recent reports of widespread infections by highly pathogenic H5N1 avian influenza virus in cattle highlight the importance of monitoring virus circulation in domestic mammals and the utility of comprehensive data-driven strategies to define the outbreak source, monitor the spread and evolution of the virus, and its pandemic potential [18]. Here, we performed a comprehensive analysis of an animal influenza epizootic at a country level. We combined genomic data of viruses collected during the FC1 EIV epizootic with epidemiological information obtained from multiple outbreak investigations. We identified multiple EIV introductions into the UK, reconstructed the dispersal history of the virus at the national scale, and identified key factors associated with regional spread. We also proposed a model of EIV international migration based on historical as well as recent data.

Our data indicate that the FC1 EIV epizootic in Europe in 2018/19 was caused by a single EIV introduction from North America, and the introduced virus was a reassortant of at least two viral lineages that circulated in the USA in the years before the epizootic. Intra-subtype reassortment has been linked to severe epidemics of H1N1 influenza in humans [38]. This, in addition to vaccine evasion, is likely to have played a central role in the high levels of transmission observed in a partially susceptible population, as horses in the UK had been exposed to different viral strains by either vaccination or natural infection. The European FC1 virus shows nonsynonymous changes in the main glycoproteins in comparison with vaccine strains. These include mutations in antigenic sites in HA; the loss of a glycosylation site -which could alter antigenicity-; and mutations in the receptor binding domain, which could alter tropism and/or infectivity. Although the impact of individual mutations remains to be determined, studies using virus neutralization tests with horse antisera suggest antigenic differences between recent FC1 strains and the vaccine strain [39,40]. Our combined data underscore the link between within-host and population-scale processes and are consistent with modelling studies showing that both the probability of infection and the size of outbreaks are positively correlated with antigenic distances between immunizing and infecting strains [30,31]. The interplay between genetic diversity, antigenic evolution, and epidemic size also applies to other RNA viruses such as SARS-CoV-2 and the rapid resurgence of infections associated with the emergence of variants of concern such as Omicron [41].

The process by which virus lineages are introduced into a region is an important aspect of early epidemic growth [32]. While our combined epidemiological and genomic data strongly support multiple virus introductions into the UK, the lack of background EIV sequences from other countries and the low genetic diversity observed in EIV genomes would have limited our ability to infer if a cluster had originated from a single introduction or multiple aggregated introductions using genomic data alone. This limitation in our sequence dataset highlights the need for enhanced genomic surveillance in Europe and globally. However, it also underscores the power of phylodynamic approaches that integrate virus epidemiology and evolution. Data from the UK surveillance system support multiple EIV introductions: Whitlock et al. [34], showed that 99 out of 234 of EI-affected premises during 2019 received new horses within two weeks before the confirmation of EI cases. Moreover, in 42 of these 99 premises the new arrivals came from European countries, with Ireland being the main country of origin associated with 36 outbreaks. Furthermore, early cases of Clusters I, IV and V were linked to horses imported from Ireland. Overall, our combined genomic and epidemiological data indicate that multiple introductions from Europe played a key role in 2018/2019 EI epizootic in the UK.

Despite the size and duration of the epizootic, most viral clusters displayed limited or no onward transmission, which could be due to different factors. For example, multiple control measures were put in place during the first phase of the epizootic when horseracing was postponed, vaccination was enhanced, and surveillance increased, mainly in sub-populations of professional horses such as competition horses [34]. As the second phase of the epizootic affected mainly horses that had no requirement of mandatory vaccination, the structure and immunological status of the population likely played a significant role in the observed burst-fade-out dynamics, which is common during outbreaks of canine influenza in dogs in the US [42]. At the geographical level, our analyses showed a complex migration pattern and a highly connected host network: three English regions (East, West Midlands, and North-West) were associated with distinct viral clusters and constituted the main sources of virus lineages at the national level. Parts of these three regions are characterised by higher horse density areas (e.g., in and around Newmarket in the East of England), where it is possible that different contact networks (e.g., training and breeding racehorses near Newmarket) may have contributed to sustained transmission chains [43]. Phylogeography-based GLM analysis showed that the number of EI-affected premises and the number of horses in local areas are predictors of further transmission to other regions. This might have been particularly relevant during the first phase with affected areas mainly located in the South and Central areas of Great Britain and where EI-infected premises classified as professional (i.e., racing, racing pre-training, training, competition or sales preparation) were more likely to be confirmed [34]. As horses are relatively easy to transport by road, they can readily travel long distances to participate in events, mixing with other horses from all over the UK, and returning to their home location on the same day [44]. In addition, EI has a short incubation period and there is potential for a horse to be infectious before displaying clinical signs and hence they can contribute to the rapid virus spread that is usually observed during large epizootics [7,45]. During the second phase of the epizootic, previously unaffected areas such as Wales, Northeast England, and Scotland reported cases, largely linked to horse movements to equine gatherings without mandatory vaccination requirements, accounting for a large number of cases and likely contributing to the virus dissemination across distant regions [34]. This is consistent with the lack of spread of a genetically similar FC1 EIV introduced in East Lothian (Scotland) in February 2018 [46]. We speculate that the low density and connectivity of the Scottish horse population could have contributed to the limited transmission of this virus. Information about horse movement, event participation, and equestrian activity are not routinely recorded as part of surveillance efforts within the UK. Future studies should aim to incorporate these data to enhance phylogeographic analyses and better understand how horse contact networks influence viral migration. With regard to control measures, our results suggest that enhanced surveillance and increased vaccination coverage in highly connected populations and populated areas, together with stricter biosecurity and vaccination requirements among highly mobile animals would be an effective strategy to prevent widespread outbreaks [47].

Finally, source-sink models have been proposed to explain the circulation patterns of various RNA viruses including SARS-CoV-2, foot and mouth disease virus [48], and IAVs in humans and swine [14,15]. According to this model, genetic and antigenic viral diversity is generated and expanded due to sustained transmission in *source* populations, which then colonise *sink* populations, with the latter characterised by higher bottlenecks and extinction rates [49,50]. Our phylogeographic analysis of global EIV sequences suggests that North America acts as the primary source of virus to Europe (and probably indirectly to the UK from European countries) and other continents driving the long-term evolution of EIV. Consistent with this, more than 30% of the global equine population is located in North America, with the US and Canada being major stakeholders in the global equine industry [51,52]. Further, as EIV is endemic in the US, its large effective population size allows natural selection to proceed more efficiently on antigenic and genetic diversity, which is further enhanced by genomic reassortment. Additional support to this view is the fact that complete EIV lineage replacements occurred in Europe in the late 1980s (seed of the Eurasian lineage), early 2000s (seed of FC2 then diverged from the FC1) and lastly in 2018 (seed of European FC1). In addition, differences in the implementation of control and prevention measures for imported horses between countries might also contribute to this asymmetric viral export from North America [23]. In conclusion, our results indicate that EI epizootics in the UK are caused by virus importations, usually followed by virus extinction. This circulation pattern suggests that endemic EIV transmission might not be supported by the UK horse population, and thus eradication of EI could potentially be achieved.

## Materials and methods

### Sequenced outbreaks and epidemiological data from the UK epizootic

During the EI epizootic that took place in the UK in 2019, a total of 234 premises were reported (confirmed through laboratory testing) to be affected [34]. Viral samples for sequencing were obtained from horse nasal or nasopharyngeal swabs with laboratory-confirmed equine influenza diagnosis by qPCR against the nucleoprotein (NP) and matrix (M) coding regions [53]. To minimize the introduction of artefact mutations during virus isolation, the viral genomic segments were PCR-amplified and sequenced directly from swabs, using the same RNA extracts obtained during diagnosis. Briefly, RNA was extracted from 200 uL of virus transport medium using the Thermo Scientific KingFisher Flex Purification System. PCR amplification was carried out by RT-PCR using universal primers as previously described [54]. Sequencing libraries were prepared as follows: up to 80 ng of each viral DNA amplicon were fragmented to a range of 300–400 bp by sonication using a Covaris Sonicator LE220. The fragmented DNA was subject to library preparation by using a KAPA Library Prep kit (KAPA Biosystems) with index tagging. NEBNext Multiplex Oligos for Illumina (Dual Index Primers Set 1 and Set 2, New England Bio-Labs, E7780S and ES7600S) was used. Libraries were quantified by Qubit (ThermoFisher) and TapeStation (Agilent) and pooled at equimolar concentrations for sequencing on the Illumina NextSeq500 platform using the NextSeq 500/550 Mid Output Kit v2.5 (2x 151Cycles). We sequenced 187 complete EIV genomes, obtained from 126/234 (~54%) premises. A single EIV genome was derived from 93 premises (93/126, 73%), and between 2 and 12 genomes were obtained for the remaining 33 (26.2%). S20 Fig shows the epizootic curve of EI in the UK, highlighting the sequenced outbreaks. The geographical and temporal distribution of the epizootic is shown in S21 Fig. Metadata for both individual confirmed cases and affected premises were collected by the former Animal Health Trust (AHT) during 2019, as detailed in [34] and summarized in S7 Table and S1 Table, respectively.

### Viral genome assembly

The raw sequencing data was processed using Trimmomatic v0.32 to eliminate adaptors, low-quality bases, and reads with lengths of less than 50 nucleotides [55]. FASTQC was used to inspect the quality of raw and post-cleaning fastq files [56]. The remaining reads were mapped against the reference sequence A/equine/Tipperary/1/2019 (GISAID isolate ID EPI_ISL_348425) using the BWA-MEM algorithm [57]. Samtools was used to index reads, calculate genome coverages, and generate BAM files [58]. Consensus sequences were called using iVar, using as a threshold a Phred score >20, position coverage >10 reads, and a minimum frequency threshold of 0.6 to call an unambiguous base [59]. Consensus genomes were submitted to GenBank under accession numbers PQ131251 to PQ132750.

### Phylogenetic and phylodynamic analyses

#### Datasets assembly, alignment, and sequence quality control.

Sequences obtained in this study and available in public databases were used to assemble different datasets according to the analysis goals. First, EIV H3N8 sequences were retrieved from the Global Initiative on Sharing All Influenza Data (GISAID) and the Influenza Virus Resource from the NCBI (last updated on April 1^st^, 2024) using the terms “Type=IAV, Subtype= H3N8, Host=mammals”. Duplicated sequences from the same isolate and redundant records between databases were eliminated based on the strain name. Sequences were sorted by viral segment and two databases were created: the complete genome dataset including viruses with the eight genomic segments sequenced (n = 436), and the HA-only dataset (n = 1280), including sequences generated in this study. A detailed distribution of sequences per region per year can be found in S8 Table.

All datasets were handled using SeqKit v.2.8 and Aliview v.1.27, aligned with MAFFT v.7.310 using the default parameters, and manually edited to eliminate the 5’ and 3’ UTRs [60–62].

IQ-TREE v.2.1 was used to estimate the molecular evolutionary models according to the Bayesian Information Criterion (BIC) statistics and to obtain Maximum likelihood trees [63,64]. The SH-like approximate likelihood ratio test (1,000 replicates) and ultrafast bootstrap approximation (1,000 replicates) were used to evaluate the reliability of the branches and groups obtained [65,66].

The congruence between the sampling date and genetic divergence was evaluated using root-to-tip regression in Tempest v1.5.1 software [67]. To detect intra-subtype reassortment, the eight genomic segments were concatenated and evaluated by Phi-test implemented in SplitsTree4 [68] as well as RDP5 [69], in addition to the visual inspection of the individual phylogeny from each genomic segment. The use of RDP can be effectively applied to detect reassortment events in segmented viruses like influenza when analyzing concatenated full genomes. In this context, a reassortment event, where a genome comprises segments derived from two parental strains, produces a sequence mosaic resembling the recombination signal in single genome organisms or chromosomes.

#### Phylodynamic reconstruction of viral spread inside the United Kingdom.

Phylogenetic and phylogeographic analyses were performed to identify internal nodes and descendent clades that likely correspond to distinct introductions into the UK and their subsequent spread. Only complete genome sequences from the UK 2018/19 epizootic generated in this study were included in this analysis. The complete genome was concatenated to maximize the phylogenetic signal, due to the low genetic divergence accrued in the individual viral genes over the short timescale. Time-scaled phylogenies, population dynamics, and geographical spread were reconstructed using BEAST v1.10.4 with the BEAGLE library to improve computational performance [70]. All analyses were run for 200 million iterations across two independent Markov chain Monte Carlo (MCMC) and samples were taken every 20,000 steps.

First, different combinations of molecular clocks (uncorrelated lognormal and strict molecular clocks) and demographic coalescent models (constant growth, exponential growth, and the non-parametric skygrid) were tested and compared using the marginal likelihood estimated by the path sampling and stepping-stones simulations. Temporal calibration was based on the tip dates and a soft prior was applied to the root height (normal distribution with mean = 0.7 years and standard deviation = 0.05) based on the distribution of the time to the more recent common ancestor (MRCA) obtained from the analyses of the full FC1 datasets, as described in the results section.

Once the best model combination was determined (uncorrelated clock and skygrid, see S9 Table), a new analysis was set up incorporating a discrete phylogeographical model. Each tip was labelled with the sampling location according to the International Territorial Level 1 region (ITL1). An asymmetric substitution matrix over the sampling locations was set up, and the Bayesian Stochastic Search Variable Selection (BSSVS) procedure was implemented. This analysis was complemented with an estimation of the expected number of migration events between all pairs of locations (Markov jumps) throughout evolutionary history [71,72], and summarised using the TaxaMarkovJumpHistoryAnalyzer tool available in the BEAST codebase (https://github.com/beast-dev/beast-mcmc). BEAST XML file used for this analysis is available as S1 File.

Tracer v1.6 was used to evaluate the convergence of parameters (i.e., effective sample size (ESS) ≥ 200, acceptable mixing without tendencies in traces, with a burn-in of 10%). The posterior distribution of samples was summarized and analysed afterwards. The Maximum clade credibility (MCC) tree was summarized using Tree Annotator v1.10.4 with the common ancestor height option and plotted using ggtree [73]. Viral clusters (I to VIII) were defined by identifying the deepest nodes in the MCC tree with high posterior probabilities (≥ 0.9). Genetic divergence among descendant viruses was not considered, and subclusters were not defined. The links between locations that contribute significantly to explaining the migration history were identified based on Bayes Factor (BF) estimation, following the methods outlined in Lemey et al. (2009) [74]. BF > 3 was considered well-supported, as proposed by [75]. Viral migration history between all supported location transitions was visualized in circular migration flow plots using the package “circlize” available in R [76].

To verify results in the discrete phylogeographic analyses and evaluate the impact of the sampling bias between the UK regions, more evenly distributed datasets were assembled by down sampling the sequences. Three approaches were applied: (i) balanced proportion of sequenced cases relative to total confirmed cases per region, where sequences were down sampled to match the lowest proportion (0.25 for London); (ii) balanced proportion of sequences outbreaks, first just one sequence per outbreak was kept, and then the remaining sequences were further down sampled to the lowest proportion of sequenced outbreaks (0.353 for South West England) relative to total outbreaks in each region; (iii) an equal number of sequences per region, where all regions were down sampled to match the number of the region with fewest sequences (three sequences in London) and, to address potential sampling bias due to the low number of sequences, ten replicated datasets were generated. Down sampling was performed in RStudio using custom scripts to randomly select the corresponding number of sequences per group. A summary of the number of sequences in the different datasets can be found in S9 Table. Analyses were run in BEAST v1.10.4 using the same models and settings as described for the full dataset. The XML files used for the analyses are available as S2–S13 Files.

Lastly, we hypothesised that the different viral clusters identified in our dataset represent independent introductions to the UK. This implies that transitions on branches connecting these clusters are incorrectly inferred as within-UK transmissions. To mitigate the impact of these basal transitions on the estimated migration matrix and highlight which transitions are dominant in the model when only the supported viral clusters are considered, we performed an additional validation analysis using a hierarchical phylogenetic model (HPM) [77,78]. This approach was previously applied to IAV datasets, both to different genes from the same set of taxa as well as independent partitions with distinct taxa sets [74,79]. Here, we implemented a HPMs where each phylogenetic cluster was introduced as a separated partition with an independent and individual relaxed clock model and tree priors while sharing a common discrete phylogeographic model. This results in a joint estimation of the transition (asymmetric) matrix for the discrete trait. Partitions representing clusters II and VI were not included since they have only two taxa. Analyses were run in BEAST v1.10.4 implementing the BSSVS procedure to reduce the number of parameters to those with significantly non-zero transition rates and BF were estimated and interpreted as for previous analyses. The XML files used for this analysis are available as S14 File.

#### Identifying correlates of viral migration.

A Generalized Linear Model (GLM) extension of the discrete trait model was used to investigate the potential contribution of region-associated variables to the viral dispersal rates. The following potential predictors were evaluated: (i) the number of horses in the premise area, (ii) the number of EI-affected premises (based on lab-confirmed cases) (iii) the proportion of sequenced cases (included to assess the effect of sample bias in the model), both at the origin and destination location, as well as predictors based on pairwise measures: (iv) a binary qualitative predictor specifying if regions share a common border, (v) the estimated driving distance between regions. Predictor matrices were built as follows. The median number of horses in the affected area was based on the model distribution of the horse population in the UK published by Lo Iacono [80]. First, only premises with post-code level location information were selected (185/234), then each premise was geolocalized within the modelled grid to determine the predicted number of horses in the corresponding area, and finally, the values were summarized using the median of the distribution for each region at the ITL1 level. The number of affected premises per region was obtained by grouping all the premises within a region that had at least one laboratory-confirmed case (S10 Table). The proportion of sequenced cases was estimated as the number of sequences over the total number of laboratory-confirmed cases per region (S10 Table). To obtain the driving distance between regions, first, the travel distance between all pairs of EI-affected premises were estimated using the Googleway R package v2.7.8 [81], and then the data were summarized to the mean of all-pair distance between each pair of regions. Analysis was run in BEAST v1.10.4 using the same models and settings and then summarized as described above. The XML file used for this analysis is available as S14 File.

#### Correlation of epidemiological traits and phylogeny.

Phylogenetic trait-association tests were performed using the BaTS (Bayesian Tip-Significance testing) package [82], to assess the clustering of the sampling location at the ITL1. A subsampled dataset was built, including only one sequence per cluster per affected premises to avoid oversampling in those facilities where more than one sequence was available. The dataset was analyzed with BEAST v1.10.4 as described for the reconstruction of viral spread inside the UK, and the posterior distribution of phylogenies obtained was used as input for the BaTS analysis. The significance of clustering was assessed by comparing the calculated association index (AI) and parsimony score (PS) from 1000 posterior samples of the trees against null distributions generated from randomizations of traits to tips.

### Analysis of the genomic evolution of Florida Clade 1 viruses

To track the source and the evolutionary history of the viral lineage that caused the 2019 European epizootics, we analysed FC1 viruses isolated since their divergence of FC2 in 2003.

#### New genomic sequences from North America.

Samples were obtained from various diagnostic centres across the United States, and submitted to the Department of Veterinary Science, University of Kentucky, USA, following routine passive diagnostic surveillance for respiratory viruses. Extracts from nasopharyngeal swabs were cultivated in embryonated hens’ eggs following standard viral isolation protocols [83]. Infected allantoid fluid was collected and shipped frozen on dry ice to the Centre for Virus Research (Glasgow, UK). Finally, RNA extraction, amplification, and sequencing were performed as described for the UK swab samples [59]. Consensus genomes were submitted to GenBank under accession numbers PQ111128 - PQ111391.

#### Florida Clade 1 dataset assembly.

Two different datasets were analysed. On one hand, a complete genome dataset was assembled to study the evolution of all the viral segments and to describe the viral dynamics before and during the 2019 European epizootics (n = 204). On the other hand, an HA-only dataset was used to provide a more geographically comprehensive dataset that incorporated genomic data from other European and Asian countries also affected during 2018/2019 where only partial genomic sequences are available. Due to the number of sequences on the later dataset, only isolates from 2016 onward were kept (n = 359). In addition, the UK sequences obtained in this study were down-sampled to balance the number of isolates in comparison with those available for other locations and years. To do this, a phylogenetically informed subsampling was performed, focused on maintaining the basic clustering patterns and time coverage, whilst reducing the noise derived from overrepresented lineages. Firstly, one sequence per cluster per outbreak was randomly selected. Additionally, highly similar sequences within the same viral cluster and collected within the same week were further downsampled, resulting in a final dataset of 74 sequences representing the UK outbreaks. A detailed breakdown of the number of sequences per year per region in both the complete genome and HA-only datasets before and after the downsampling can be found in S11 Table. All datasets were handled and examined as described above, and maximum likelihood trees were obtained with IQ-TREE for each dataset accordingly.

#### Phylodynamic reconstruction of Florida Clade 1 evolution.

First, the HA alignment from the complete genome dataset was used to evaluate and select the best-fit clock and demographic models by comparing the marginal likelihood (S12 Table). The selected combination (strict clock and skygrid models) was then applied to all the remaining segments. In addition, sequences were tagged with their geographical region of origin (i.e., Africa, Asia, Europe, North America and South America), an asymmetric substitution matrix over the sampling locations and the BSSVS procedure were implemented to incorporate a discrete phylogeographic model. Individual time-calibrated phylogenies, evolutionary rates and population dynamics were co-estimated in BEAST v1.10.4. Analyses were run for 200 million iterations and samples were taken every 20,000 steps and summarized as described before. The XML files used for the analyses are available as S16–S24 Files.

### Source-sink model for EIV global dynamics

We aimed to identify the global transmission patterns in the long-term evolution of EIV. Thus, the HA-only dataset including isolates from 1963 to 2022 was used. Each sequence was tagged with the region of origin (North America, South America, Europe, Asia, and Africa) and a subsampling to eliminate identical sequences per region per year was performed, to reduce redundancy and the size of the data frame. Sequences from the outbreaks that occurred in Australia in 2007 were not included since excluding the epizootic, Oceania has been free of EI for decades and thus does not contribute to EIV dynamics. The final dataset includes a total of 698 sequences (a detailed number of sequences used per region per year can be found in S8 Table). Phylodynamic analyses were run in BEAST v1.10.4 setting up a strict clock using tip-dates temporal calibration, a skygrid model, an asymmetric substitution matrix over the sampling locations with the BSSVS procedure, the Markov jump estimation of the number of location transitions. Analyses were run for 200 million iterations and samples were taken every 20,000 steps and summarized as described above. The XML file used for this analysis is available as S24 File.

Validation analyses using down sampled datasets were run to evaluate the impact of the sampling bias across the different global regions, mainly the overrepresentation of sequences from Europe and North America. Down sampled datasets were assembled as follows: sequences were grouped in the categories North America, Europe and Others (including South America, Africa and Asia). Then, two different strategies were applied: (i) balanced number of sequences per region per year: the number of sequences in each region was down sampled to the minimum number of sequences in a region in each year; (ii) balanced number of sequences per region per period: four intervals were considered, reflecting different periods in the EIV phylogeny and the circulation of different lineages, as follows: 1963–1984 including the “pre-divergent” strains; 1985–2001 including the circulation of Eurasian, Kentucky and South America Lineages; 2002–2017 predominance of the Florida Clade 1 and 2; 2018–2022 dominance of Florida Clade 1. Sequences were down sampled to match the lowest count in a region in each period. A summary of the number of sequences in the different datasets can be found in S8 Table. Analyses were run in BEAST v1.10.4 using the same models and settings as described for the original dataset. The XML files used for the analyses are available as S26 and S27 Files.

### Data analysis and statistics

All data were handled, analysed, and plotted in R v 4.4.1 using RStudio with packages including tidyversev, rstatix, ggplot2, rnaturalearth. Phylogenetic trees were plotted using ggtree v 3.12.0.

A linear mixed-effects model was used to analyze the impact of the vaccination status and the time since symptom onset on the viral load. The analysis was performed using the R statistical software v 4.4.1. The linear mixed-effects model was fitted using the lmer function from the lme4 package v1.1-35.5 along with the lmerTest package v 3.1-3. The independent variable was introduced as the log10 of copy number for M qPCR values (data in S7 Table). The fixed effects in the model included the vaccination status (only horses with the status “vaccinated” or “unvaccinated” were included) and a squared term for days since signs onset and sampling to account for the non-linear relation with the viral load. The model also included a random effect to account for repeated measures on the same premises. The significance of the fixed effects was evaluated using t-tests with Satterthwaite’s method to approximate degrees of freedom.

## Supporting information

S1 Fig Florida Clade 1 Maximum likelihood tree for the PB2 segment. Maximum Likelihood tree of the PB2 segment dataset generated using IQTree software. Branch values represent ultrafast bootstrap values (>95) from 1000 pseudoreplicates. Tips are coloured by sampling location. The representative parental isolates of the Europe FC1 epizootic viruses are highlighted with thicker lines, including A/equine/Idaho/1/2018 (major backbone) and A/equine/Washington/2/2018 (HA donor, marked with an asterisk).
(TIFF)

S2 Fig Florida Clade 1 Maximum likelihood tree for the PB1 segment. Maximum Likelihood tree of the PB1 segment dataset generated using IQTree software. Branch values represent ultrafast bootstrap values (>95) from 1000 pseudoreplicates. Tips are coloured by sampling location. The representative parental isolates of the Europe FC1 epizootic viruses are highlighted with thicker lines, including A/equine/Idaho/1/2018 (major backbone) and A/equine/Washington/2/2018 (HA donor, marked with an asterisk).
(TIFF)

S3 Fig Florida Clade 1 Maximum likelihood tree for the PA segment. Maximum Likelihood tree of the PA segment dataset generated using IQTree software. Branch values represent ultrafast bootstrap values (>95) from 1000 pseudoreplicates. Tips are coloured by sampling location. The representative parental isolates of the Europe FC1 epizootic viruses are highlighted with thicker lines, including A/equine/Idaho/1/2018 (major backbone) and A/equine/Washington/2/2018 (HA donor, marked with an asterisk).
(TIFF)

S4 Fig Florida Clade 1 Maximum likelihood tree for the HA segment. Maximum Likelihood tree of the HA segment dataset generated using IQTree software. Branch values represent ultrafast bootstrap values (>95) from 1000 pseudoreplicates. Tips are coloured by sampling location. The representative parental isolates of the Europe FC1 epizootic viruses are highlighted with thicker lines, including A/equine/Idaho/1/2018 (major backbone) and A/equine/Washington/2/2018 (HA donor, marked with an asterisk).
(TIFF)

S5 Fig Florida Clade 1 Maximum likelihood tree for the NP segment. Maximum Likelihood tree of the NP segment dataset generated using IQTree software. Branch values represent ultrafast bootstrap values (>95) from 1000 pseudoreplicates. Tips are coloured by sampling location. The representative parental isolates of the Europe FC1 epizootic viruses are highlighted with thicker lines, including A/equine/Idaho/1/2018 (major backbone) and A/equine/Washington/2/2018 (HA donor, marked with an asterisk).
(TIFF)

S6 Fig Florida Clade 1 Maximum likelihood tree for the NA segment. Maximum Likelihood tree of the NA segment dataset generated using IQTree software. Branch values represent ultrafast bootstrap values (>95) from 1000 pseudoreplicates. Tips are coloured by sampling location. The representative parental isolates of the Europe FC1 epizootic viruses are highlighted with thicker lines, including A/equine/Idaho/1/2018 (major backbone) and A/equine/Washington/2/2018 (HA donor, marked with an asterisk).
(TIFF)

S7 Fig Florida Clade 1 Maximum likelihood tree for the MP segment. Maximum Likelihood tree of the MP segment dataset generated using IQTree software. Branch values represent ultrafast bootstrap values (>95) from 1000 pseudoreplicates. Tips are coloured by sampling location. The representative parental isolates of the Europe FC1 epizootic viruses are highlighted with thicker lines, including A/equine/Idaho/1/2018 (major backbone) and A/equine/Washington/2/2018 (HA donor, marked with an asterisk).
(TIFF)

S8 Fig Florida Clade 1 Maximum likelihood tree for the NS segment. Maximum Likelihood tree of the NS segment dataset generated using IQTree software. Branch values represent ultrafast bootstrap values (>95) from 1000 pseudoreplicates. Tips are coloured by sampling location. The representative parental isolates of the Europe FC1 epizootic viruses are highlighted with thicker lines, including A/equine/Idaho/1/2018 (major backbone) and A/equine/Washington/2/2018 (HA donor, marked with an asterisk).
(TIFF)

S9 Fig Comparison of the Florida Clade 1 phylogenies across different genomic segments. Maximum likelihood trees for the HA (left) and NA (right) segments are shown as representatives for the other genomic segments, with lines connecting the corresponding isolates across the trees. The closest North American viruses in the HA and NA segments (A/equine/Idaho/1/2018 and A/equine/Washington/2/2018, respectively) the to the European FC1 epizootic viruses are shown with red arrows and connected with dotted lines, showing that these two viruses belong to different clusters in the individual phylogenies (consistent with reassortment). The trees are the same as presented in S4 and S6 Figs.
(TIFF)

S10 Fig Maximum Likelihood tree of the HA-only dataset of FC1. Maximum Likelihood tree obtained from the analysis of the HA-only dataset using IQTree. Values on branches represent ultrafast bootstrap values (>95), obtained from 1000 pseudoreplicates. Tips are coloured according to Location: “Countries” for European isolates and “Region” for other continents.
(TIFF)

S11 Fig Time-resolved phylogeny of UK viruses with the summary of the phylogeographic ancestral state reconstruction. MCC tree obtained from the discrete phylogeographic analysis (shown in Figs 2 and 3A). Colours on tips names and branches correspond to the regions outlined in the lower-left inset. Tips names include the designated viral cluster, outbreak ID, isolate name, ITL 1 region and collection date. Nodes with posterior probability ≥ 0.9 are labelled with diamonds.
(TIFF)

S12 Fig Contribution of each UK region to seeding viral lineages in other locations throughout the epizootic. The values represent the cumulative Markov jump counts per day from each UK region to (any) other locations, summarized as the median with 95% HPD intervals per tree across the posterior distribution. (A) Cumulative number of transitions for the three main source regions: East, West Midlands, and North West England. (B) Separate panels displaying the cumulative number of transitions for each UK region independently.
(TIFF)

S13 Fig Viral import and export for each region through time. The values represent the mean of the Markov jump count per day from (in red) and to (in blue) each region. The y-axis between the facets is not drawn to scale to enhance the visualization of the curves in regions with low jump counts. The values in the y-axis was calculated as mean number of the Markov jump per day across all trees in the posterior distribution and smoothed using a 7-day centred rolling mean.
(TIF)

S14 Fig Markov reward times per region. The boxplot of each region depicts the density distribution of the total time spent (boxes show the median and HPD80 interval).
(TIFF)

S15 Fig Estimated proportion of transition events between UK regions using balanced datasets. (A) Results from the full dataset are compared to datasets down sampled by the number of confirmed cases and by the number of outbreaks or affected premises per region. (B) Results from replicates (n = 10) of datasets down sampled to an equal number of sequences per region. The heatmap colours represent the proportions of between-region Markov jumps relative to the total number of jumps in each analysis. Links supported by Bayes factors (BF) are highlighted by black (≥3) or red (≥20) edges.
(TIF)

S16 Fig Predictors of viral migration rates between UK regions. The boxplot represents the contributions of each predictor when included in the model (boxes show the median and HPD80 interval). The BF associated with each predictor considered in the GLM are reported on the right, with supported ones (BF ≥ 3) labelled with an asterisk.
(PNG)

S17 Fig Percentage of total migration Markov jumps counts from a region to each region during the whole evolution of the H3N8 EIV lineage. Markov jumps counts obtained from the phylodynamic analysis in BEAST. The bars represent the percentage of the total Markov Jumps count from (solid-coloured bars) and to (transparent bars) each region.
(TIFF)

S18 Fig Viral import and export for each region through time during the evolution of the H3N8 EIV lineage. Markov jumps counts obtained from the phylodynamic analysis in BEAST. The values represent the median of the Markov jump count per year “from” (positive y-axis) and “to” (negative y-axis) each region.
(TIFF)

S19 Fig Estimated proportion of transition events between global regions using balanced datasets. Comparison of proportions of between-region Markov jumps counts and rewards in the (i) original dataset, (B) the balanced number of sequences per region per year and, (C) balanced number of sequences per region per phylogenetic period.
(TIFF)

S20 Fig Epizootic curve of affected premises in the UK. Epizootic curve of affected premises reported per epidemiological week and the corresponding sequencing status. Shaded areas indicate the first and second epizootic phases as defined in [35].
(TIFF)

S21 Fig Geographical distribution of the affected premises during the epizootic in the UK. Each circle corresponds to a lab-confirmed facility coloured according to the viral cluster identified. Base UK map shapefile sourced from Natural Earth (https://www.naturalearthdata.com/) public domain.
(TIFF)

S1 Table Epidemiological data of the EI affected premises during the 2018–2019 epizootic in the UK. Metadata for individual confirmed cases was collected by the former Animal Health Trust (AHT), as detailed in Whitlock et al (2023) [35]. Sequencing status and viral clusters detected correspond to the information added in this study.
(XLSX)

S2 Table Summary of association index from BaTS analysis. AI: association index; PS: parsimony score; MC: Monophyletic Clade size statistic; CI: confidence interval.
(DOCX)

S3 Table Overview of the well-supported transitions between the UK regions. The BF were obtained using a model averaging procedure (BSSVS) in BEAST and only transitions with positive or strong support (BF ≥ 3) are shown, sorted from high to low.
(DOCX)

S4 Table Inclusion support statistics for predictors evaluated in the GLM analysis. Bayes Factor (BF) was calculated with a prior odds of 0.09051 (prior probability distribution = 0.083). Predictors included in the model (BF ≥ 3) are indicated with an asterisk.
(DOCX)

S5 Table Substitution on the HA protein. Amino acid changes on the protein between A/equine/Lincolnshire/00620/2019 (UK/2019) and the vaccine strains commonly used: A/equine/South Africa/4/2003 (SA/2003) and A/equine/Richmond/1/2007 (UK/2007), and a more recent FC2 strain A/equine/Kent/2015 (UK/2015). Changes in antigenic sites, receptor binding domains (RBD) and changes in N-linked glycosylation sites are shown. Changes unique to UK/2019 are highlighted in bold.
(XLSX)

S6 Table Summary of Results of Linear Mixed-Effects Model. Df: degrees of freedom; t value: t-statistic value; Pr(>|t|): p-value with significance level codes (***: p < 0.001).
(DOCX)

S7 Table Epidemiological data of the EI confirmed cases during the 2018–2019 epizootic in the UK. Metadata for individual confirmed cases was collected by the former Animal Health Trust (AHT), as detailed in [35]. Sequencing status and viral cluster correspond to the information added in this study.
(XLSX)

S8 Table EIV H3N8 global dataset composition. Distribution of sequences per year per region in the HA datasets, including (i) all the sequences available in public databases and (ii) the number of sequences in the different down sampled datasets used in the analyses of the source-sink model for EIV global dynamics. New sequences from the USA and the UK reported in this study are indicated in bold (included in the total number).
(XLSX)

S9 Table Marginal likelihood estimation and Bayes Factor (BF) comparison for the UK 2019 dataset. Maximum likelihood estimation (MLE) was obtained for combinations of molecular clocks and demographic models using the Path sampling (PS) and Stepping-stone (SS) sampling and compared using Bayes Factors (only comparison of the PS-MLE are shown as illustrative). Bayes Factor (BF) should be interpreted as how many times the model in the column fits better to the data than the model in the row, according to Kass & Raftery, 1995 [73].
(XLSX)

S10 Table Number of sequences, confirmed cases and outbreaks in the UK regions. Total confirmed cases by qPCR during the 2018/2019 epizootic and number of outbreaks (affected premises) per region. The number of sequences in the down sampled dataset used in the validation analyses are provided in the columns “Balanced proportion of sequenced cases” (number of sequences down sampled to a proportion of 0.25, corresponding to the lowest sequencing coverage, found in the region of London) and “Balanced proportion of sequenced outbreaks” (number of sequenced outbreaks down sampled to a proportion of 0.353, corresponding to the lowest proportion of sequenced outbreaks, found in the South West England region).
(XLSX)

S11 Table Florida Clade 1 dataset composition. Distribution of sequences per year per region in the complete genome (CG) and HA-only datasets (only isolates from 2016 onward). For Europe, the number of sequences from the UK collected in 2019 included in the down sampled dataset is reported in a separated column. New sequences from the USA and the UK reported in this study are indicated in bold (included in the total number).
(XLSX)

S12 Table Marginal likelihood estimation and Bayes Factor (BF) comparison for the Florida Clade 1 dataset. Maximum likelihood estimation (MLE) was obtained for combinations of molecular clocks and demographic models using Path sampling (PS) and Stepping-stone (SS) sampling and compared using Bayes Factors (only comparison of the PS-MLE are shown as illustrative). Bayes Factor (BF) should be interpreted as how many times the model in the column fits better to the data than the model in the row, according to Kass & Raftery, 1995 [73].
(XLSX)

S1 File XML file for running the discrete phylogeographic analysis of the UK 2019 complete genome dataset in BEAST. (XML)

S2 File XML file for running the discrete phylogeographic analysis in BEAST using the of the UK 2019 complete genome dataset balanced by number of cases per region. (XML)

S3 File XML file for running the discrete phylogeographic analysis in BEAST using the of the UK 2019 complete genome dataset balanced by number of outbreaks per region. (XML)

S4 File XML file for running the discrete phylogeographic analysis in BEAST using the UK 2019 complete genome Dataset with an equal number of sequences per region (replicate 1). (XML)

S5 File XML file for running the discrete phylogeographic analysis in BEAST using the UK 2019 complete genome Dataset with an equal number of sequences per region (replicate 2). (XML)

S6 File XML file for running the discrete phylogeographic analysis in BEAST using the UK 2019 complete genome Dataset with an equal number of sequences per region (replicate 3). (XML)

S7 File XML file for running the discrete phylogeographic analysis in BEAST using the UK 2019 complete genome Dataset with an equal number of sequences per region (replicate 4). (XML)

S8 File XML file for running the discrete phylogeographic analysis in BEAST using the UK 2019 complete genome Dataset with an equal number of sequences per region (replicate 5). (XML)

S9 File XML file for running the discrete phylogeographic analysis in BEAST using the UK 2019 complete genome Dataset with an equal number of sequences per region (replicate 6). (XML)

S10 File XML file for running the discrete phylogeographic analysis in BEAST using the UK 2019 complete genome Dataset with an equal number of sequences per region (replicate 7). (XML)

S11 File XML file for running the discrete phylogeographic analysis in BEAST using the UK 2019 complete genome Dataset with an equal number of sequences per region (replicate 8). (XML)

S12 File XML file for running the discrete phylogeographic analysis in BEAST using the UK 2019 complete genome Dataset with an equal number of sequences per region (replicate 9). (XML)

S13 File XML file for running the discrete phylogeographic analysis in BEAST using the UK 2019 complete genome Dataset with an equal number of sequences per region (replicate 10). (XML)

S14 File XML file for running the discrete phylogeographic analysis of the UK 2019 complete genome dataset using a hierarchical phylogenetic model (HPM) in BEAST. (XML)

S15 File XML file for running the GLM extension of the phylogeographic discrete analysis in BEAST to identify predictors of viral migration rates between UK regions. (XML)

S16 File XML file for the phylodynamic reconstruction of Florida Clade 1 evolution using the HA-only [2016–2021] dataset. (XML)

S17 File XML file for the phylodynamic reconstruction of Florida Clade 1 evolution using the PB2 genomic segment dataset [2016–2021]. (XML)

S18 File XML file for the phylodynamic reconstruction of Florida Clade 1 evolution using the PB1 genomic segment dataset [2016–2021]. (XML)

S19 File XML file for the phylodynamic reconstruction of Florida Clade 1 evolution using the PA genomic segment dataset [2016–2021]. (XML)

S20 File XML file for the phylodynamic reconstruction of Florida Clade 1 evolution using the HA genomic segment dataset [2016–2021]. (XML)

S21 File XML file for the phylodynamic reconstruction of Florida Clade 1 evolution using the NP genomic segment dataset [2016–2021]. (XML)

S22 File XML file for the phylodynamic reconstruction of Florida Clade 1 evolution using the NA genomic segment dataset [2016–2021]. (XML)

S23 File XML file for the phylodynamic reconstruction of Florida Clade 1 evolution using the MP genomic segment dataset [2016–2021]. (XML)

S24 File XML file for the phylodynamic reconstruction of Florida Clade 1 evolution using the NS genomic segment dataset [2016–2021]. (XML)

S25 File XML file for the global migration dynamics of EIV (H3N8). (XML)

S26 File XML file for running the the global migration dynamics of EIV using the the dataset including a balanced number of sequences per region per year. (XML)

S27 File XML file for running the the global migration dynamics of EIV using the dataset including a balanced number of sequences per region per period. (XML)
