# Supplementary figures and images for: Multiple introductions of equine influenza virus into the United Kingdom resulted in widespread outbreaks and lineage replacement
Source: PLoS Pathog. 2025 Jun 9;21(6):e1013227. doi: 10.1371/journal.ppat.1013227 (PMC12236680; doi:10.1371/journal.ppat.1013227)

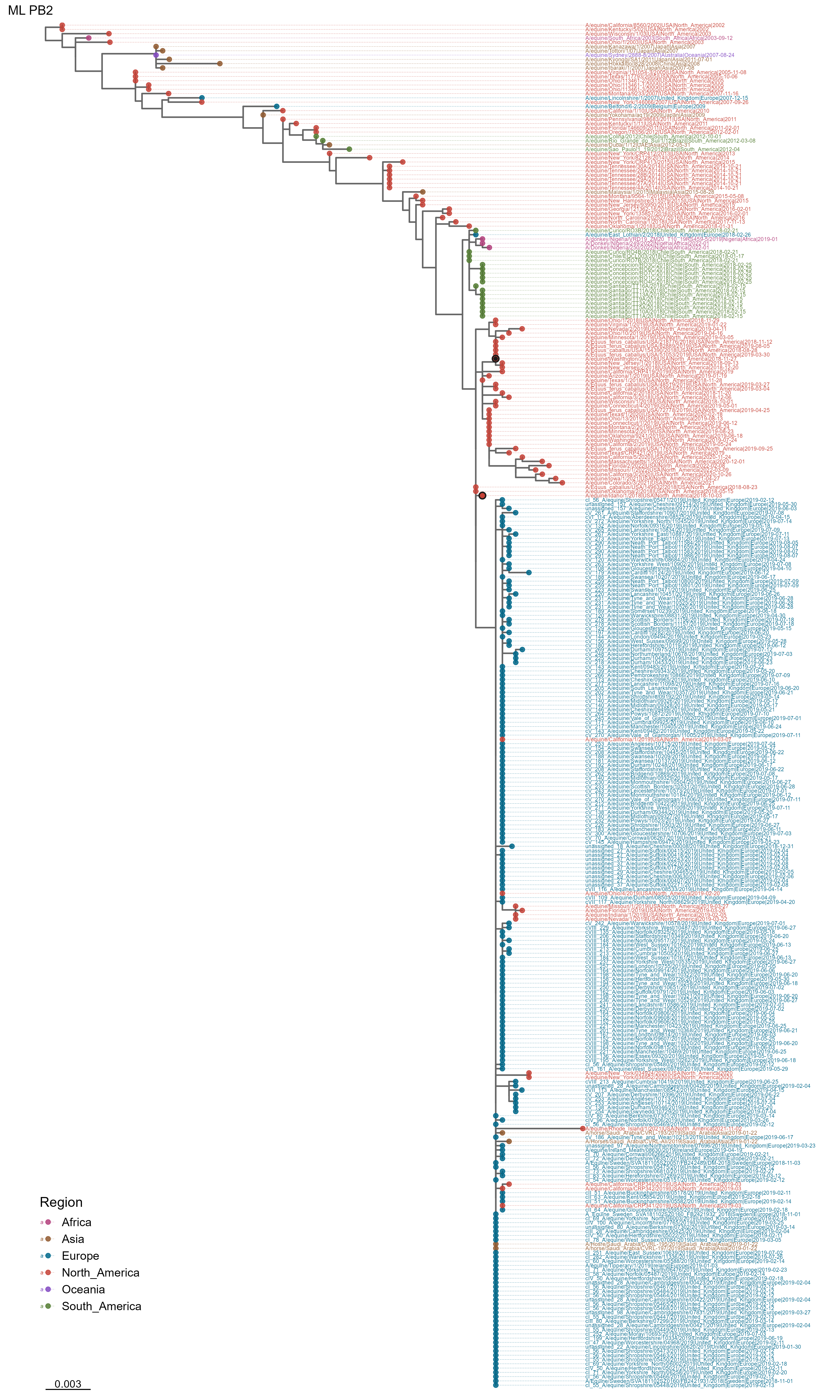

Supplement: S1 Fig — Maximum Likelihood tree of the PB2 segment dataset generated using IQTree software. Branch values represent ultrafast bootstrap values (>95) from 1000 pseudoreplicates. Tips are coloured by sampling location. The representative parental isolates of the Europe FC1 epizootic viruses are highlighted with thicker lines, including A/equine/Idaho/1/2018 (major backbone) and A/equine/Washington/2/2018 (HA donor, marked with an asterisk). (TIFF) [file ppat.1013227.s001.tiff]

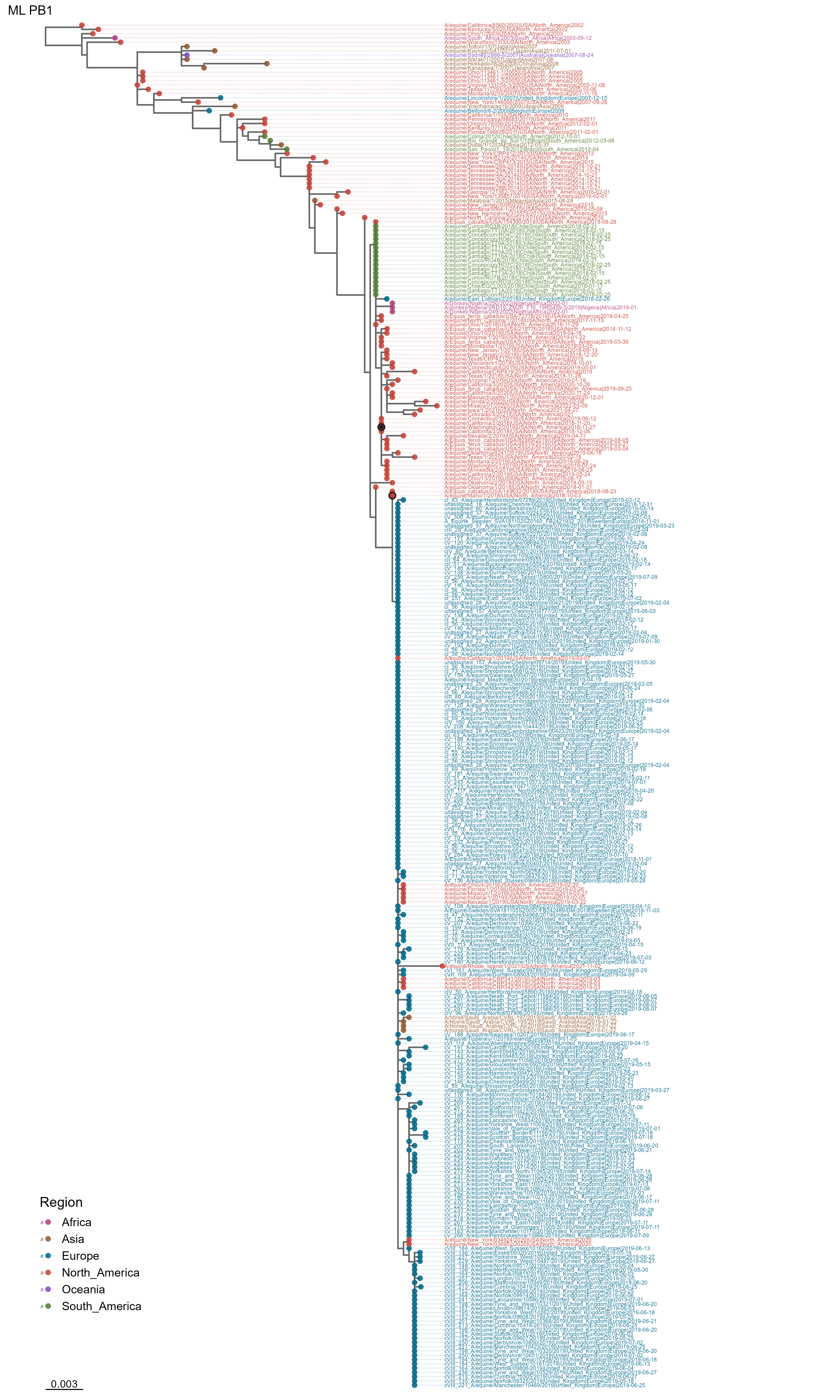

Supplement: S2 Fig — Maximum Likelihood tree of the PB1 segment dataset generated using IQTree software. Branch values represent ultrafast bootstrap values (>95) from 1000 pseudoreplicates. Tips are coloured by sampling location. The representative parental isolates of the Europe FC1 epizootic viruses are highlighted with thicker lines, including A/equine/Idaho/1/2018 (major backbone) and A/equine/Washington/2/2018 (HA donor, marked with an asterisk). (TIFF) [file ppat.1013227.s002.tiff]

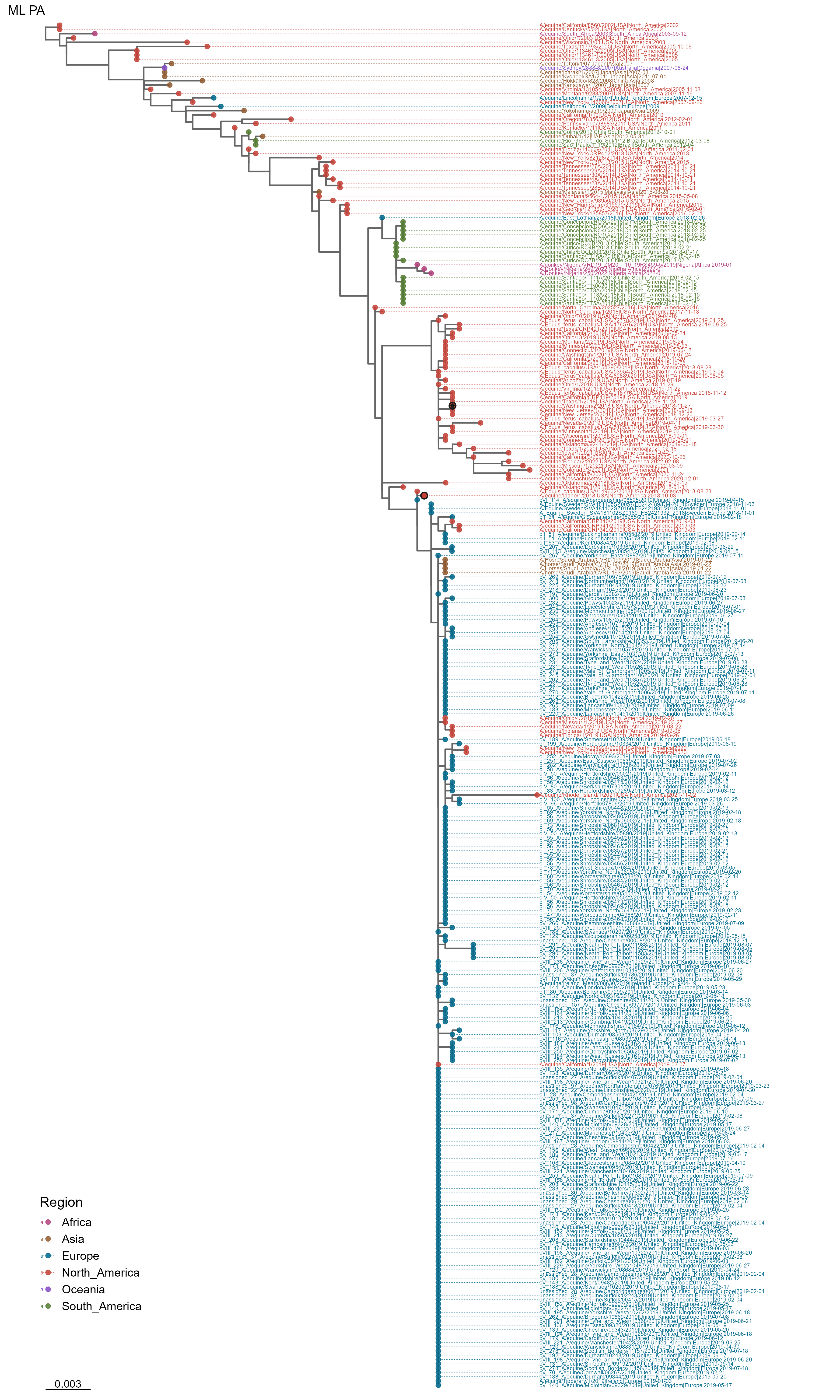

Supplement: S3 Fig — Maximum Likelihood tree of the PA segment dataset generated using IQTree software. Branch values represent ultrafast bootstrap values (>95) from 1000 pseudoreplicates. Tips are coloured by sampling location. The representative parental isolates of the Europe FC1 epizootic viruses are highlighted with thicker lines, including A/equine/Idaho/1/2018 (major backbone) and A/equine/Washington/2/2018 (HA donor, marked with an asterisk). (TIFF) [file ppat.1013227.s003.tiff]

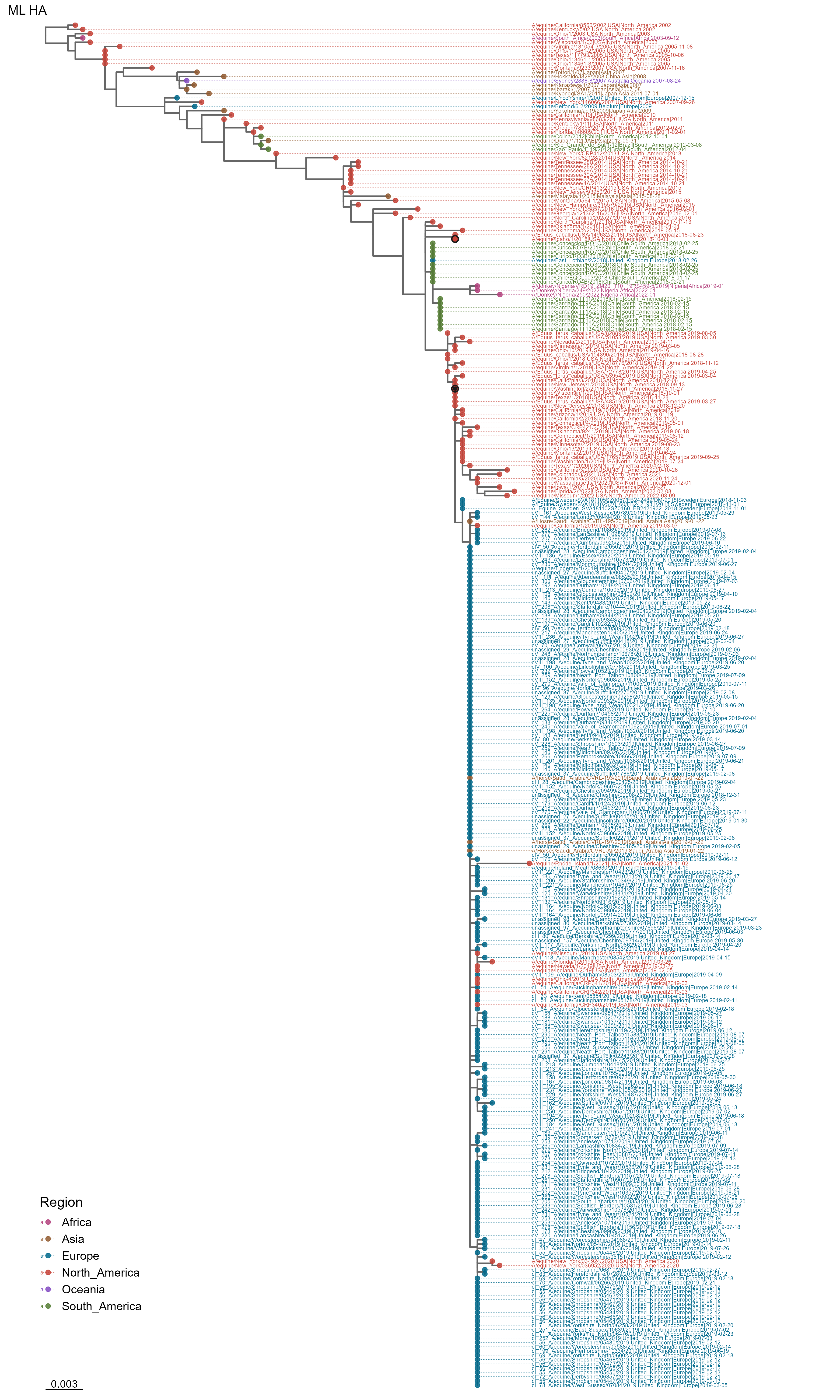

Supplement: S4 Fig — Maximum Likelihood tree of the HA segment dataset generated using IQTree software. Branch values represent ultrafast bootstrap values (>95) from 1000 pseudoreplicates. Tips are coloured by sampling location. The representative parental isolates of the Europe FC1 epizootic viruses are highlighted with thicker lines, including A/equine/Idaho/1/2018 (major backbone) and A/equine/Washington/2/2018 (HA donor, marked with an asterisk). (TIFF) [file ppat.1013227.s004.tiff]

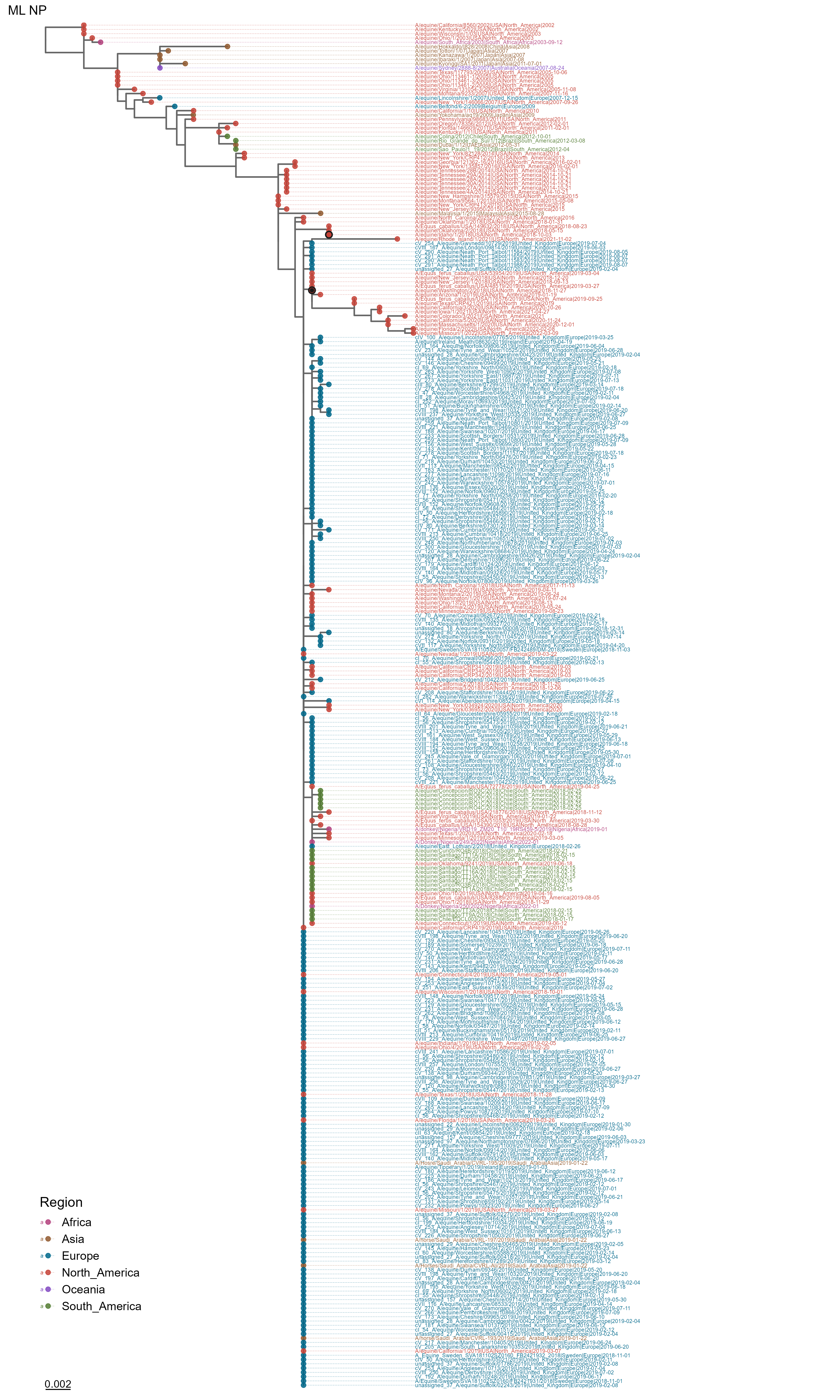

Supplement: S5 Fig — Maximum Likelihood tree of the NP segment dataset generated using IQTree software. Branch values represent ultrafast bootstrap values (>95) from 1000 pseudoreplicates. Tips are coloured by sampling location. The representative parental isolates of the Europe FC1 epizootic viruses are highlighted with thicker lines, including A/equine/Idaho/1/2018 (major backbone) and A/equine/Washington/2/2018 (HA donor, marked with an asterisk). (TIFF) [file ppat.1013227.s005.tiff]

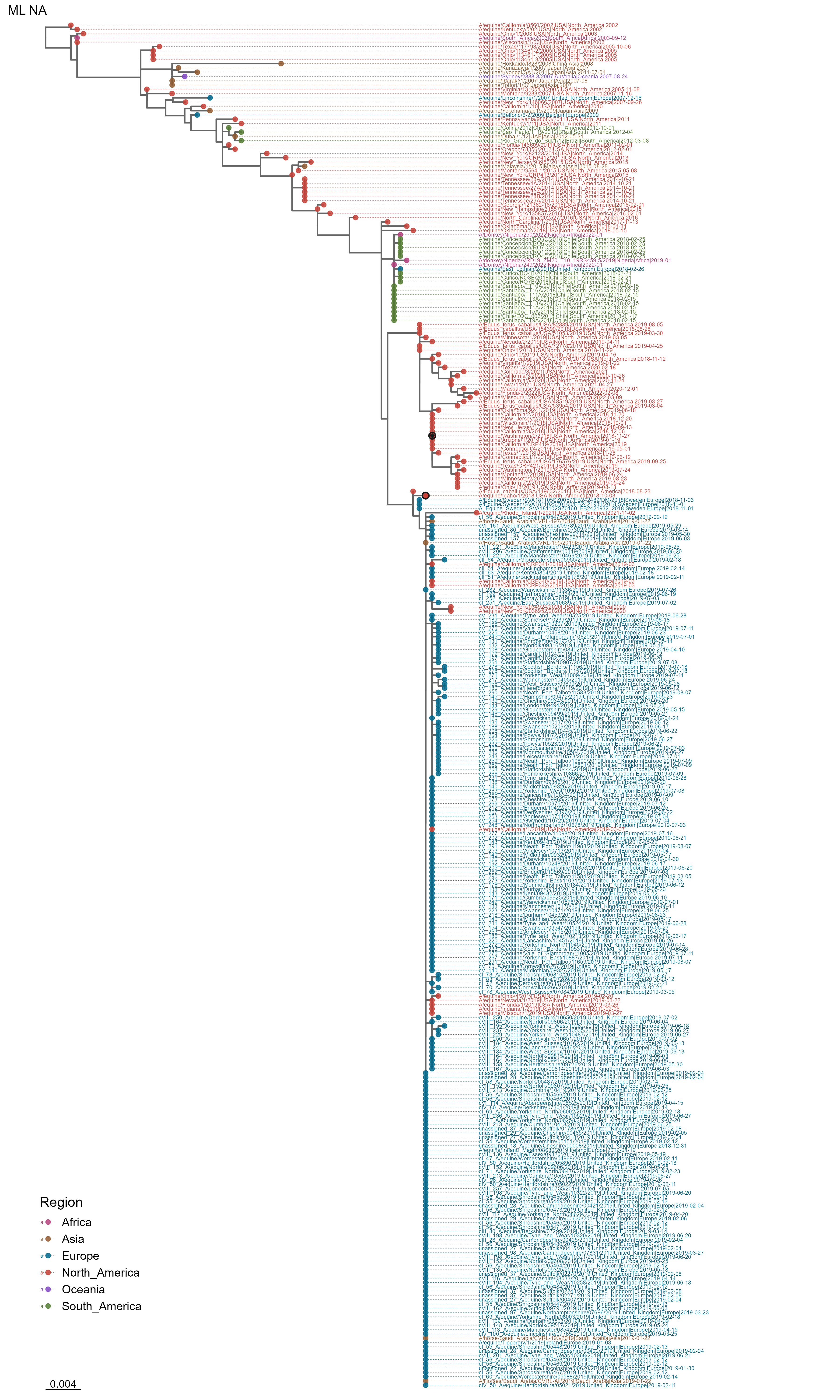

Supplement: S6 Fig — Maximum Likelihood tree of the NA segment dataset generated using IQTree software. Branch values represent ultrafast bootstrap values (>95) from 1000 pseudoreplicates. Tips are coloured by sampling location. The representative parental isolates of the Europe FC1 epizootic viruses are highlighted with thicker lines, including A/equine/Idaho/1/2018 (major backbone) and A/equine/Washington/2/2018 (HA donor, marked with an asterisk). (TIFF) [file ppat.1013227.s006.tiff]

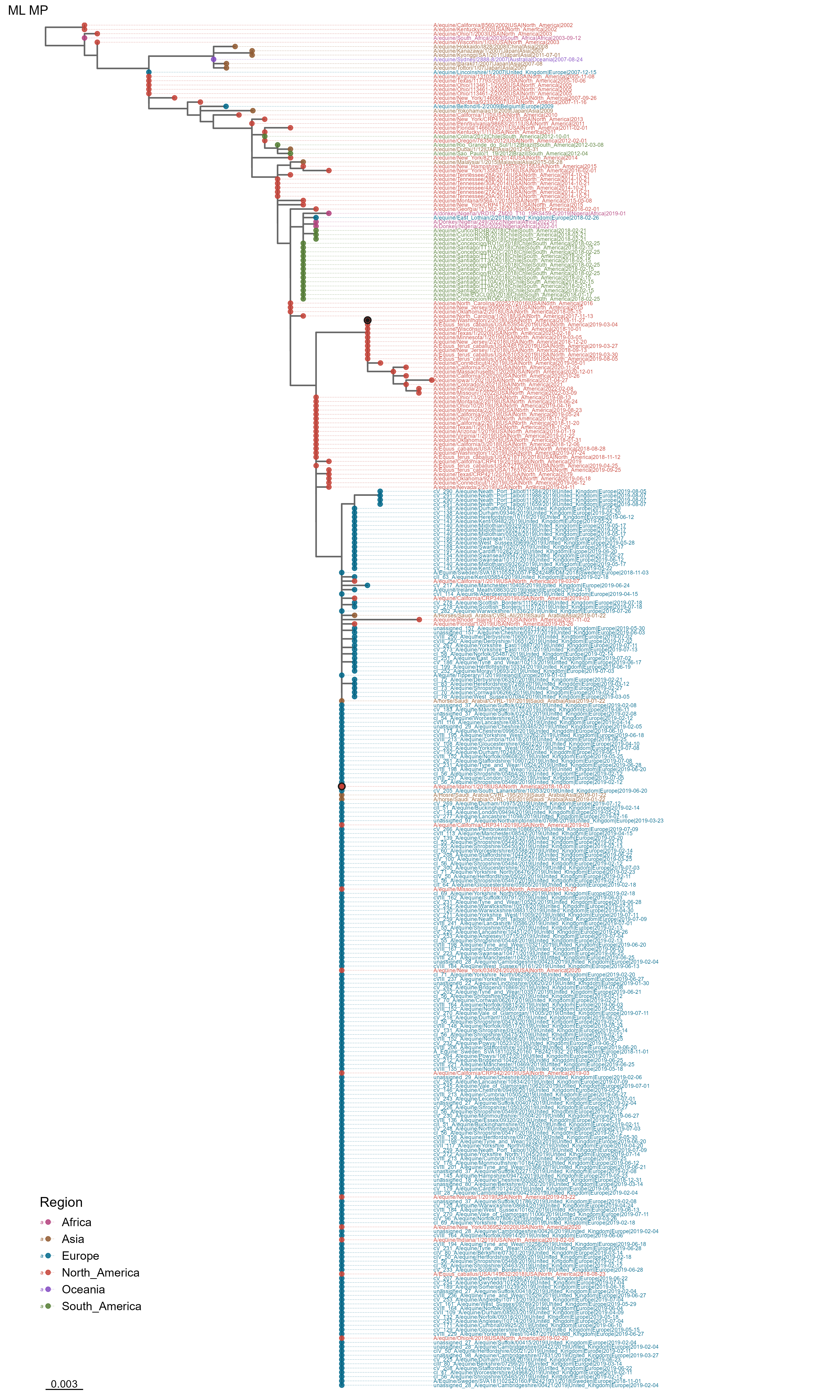

Supplement: S7 Fig — Maximum Likelihood tree of the MP segment dataset generated using IQTree software. Branch values represent ultrafast bootstrap values (>95) from 1000 pseudoreplicates. Tips are coloured by sampling location. The representative parental isolates of the Europe FC1 epizootic viruses are highlighted with thicker lines, including A/equine/Idaho/1/2018 (major backbone) and A/equine/Washington/2/2018 (HA donor, marked with an asterisk). (TIFF) [file ppat.1013227.s007.tiff]

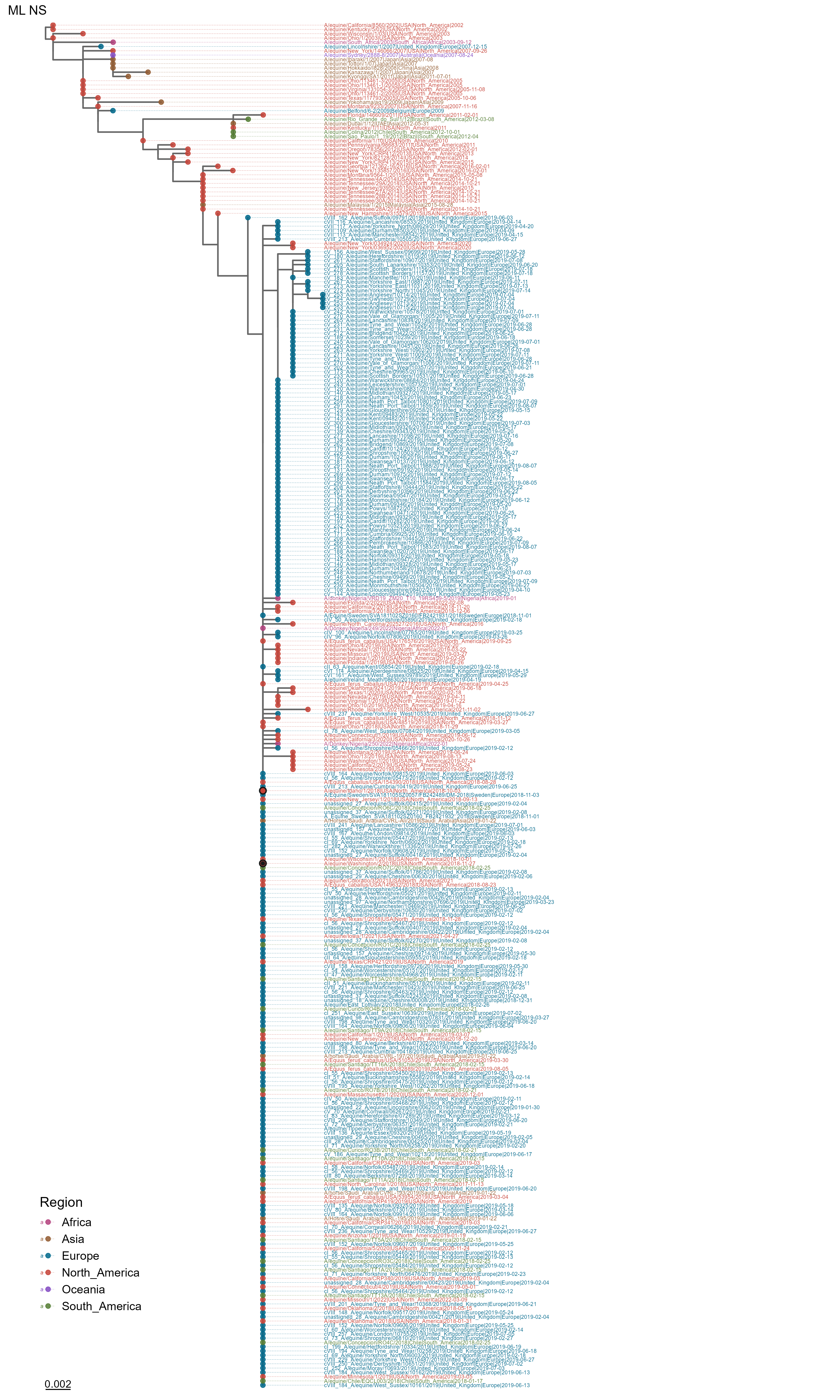

Supplement: S8 Fig — Maximum Likelihood tree of the NS segment dataset generated using IQTree software. Branch values represent ultrafast bootstrap values (>95) from 1000 pseudoreplicates. Tips are coloured by sampling location. The representative parental isolates of the Europe FC1 epizootic viruses are highlighted with thicker lines, including A/equine/Idaho/1/2018 (major backbone) and A/equine/Washington/2/2018 (HA donor, marked with an asterisk). (TIFF) [file ppat.1013227.s008.tiff]

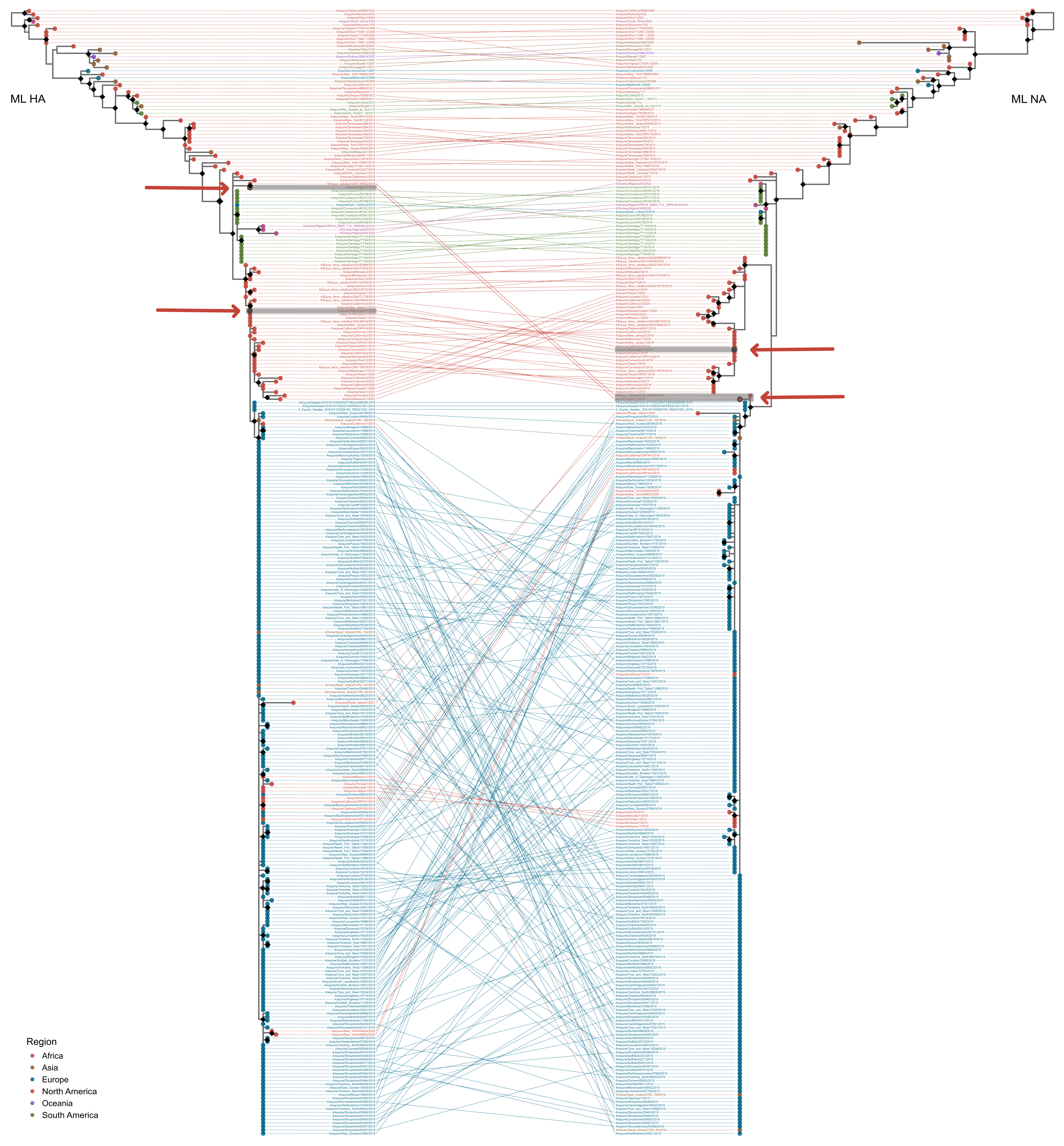

Supplement: S9 Fig — Maximum likelihood trees for the HA (left) and NA (right) segments are shown as representatives for the other genomic segments, with lines connecting the corresponding isolates across the trees. The closest North American viruses in the HA and NA segments (A/equine/Idaho/1/2018 and A/equine/Washington/2/2018, respectively) the to the European FC1 epizootic viruses are shown with red arrows and connected with dotted lines, showing that these two viruses belong to different clusters in the individual phylogenies (consistent with reassortment). The trees are the same as presented in S4 and S6 Figs. (TIFF) [file ppat.1013227.s009.tiff]

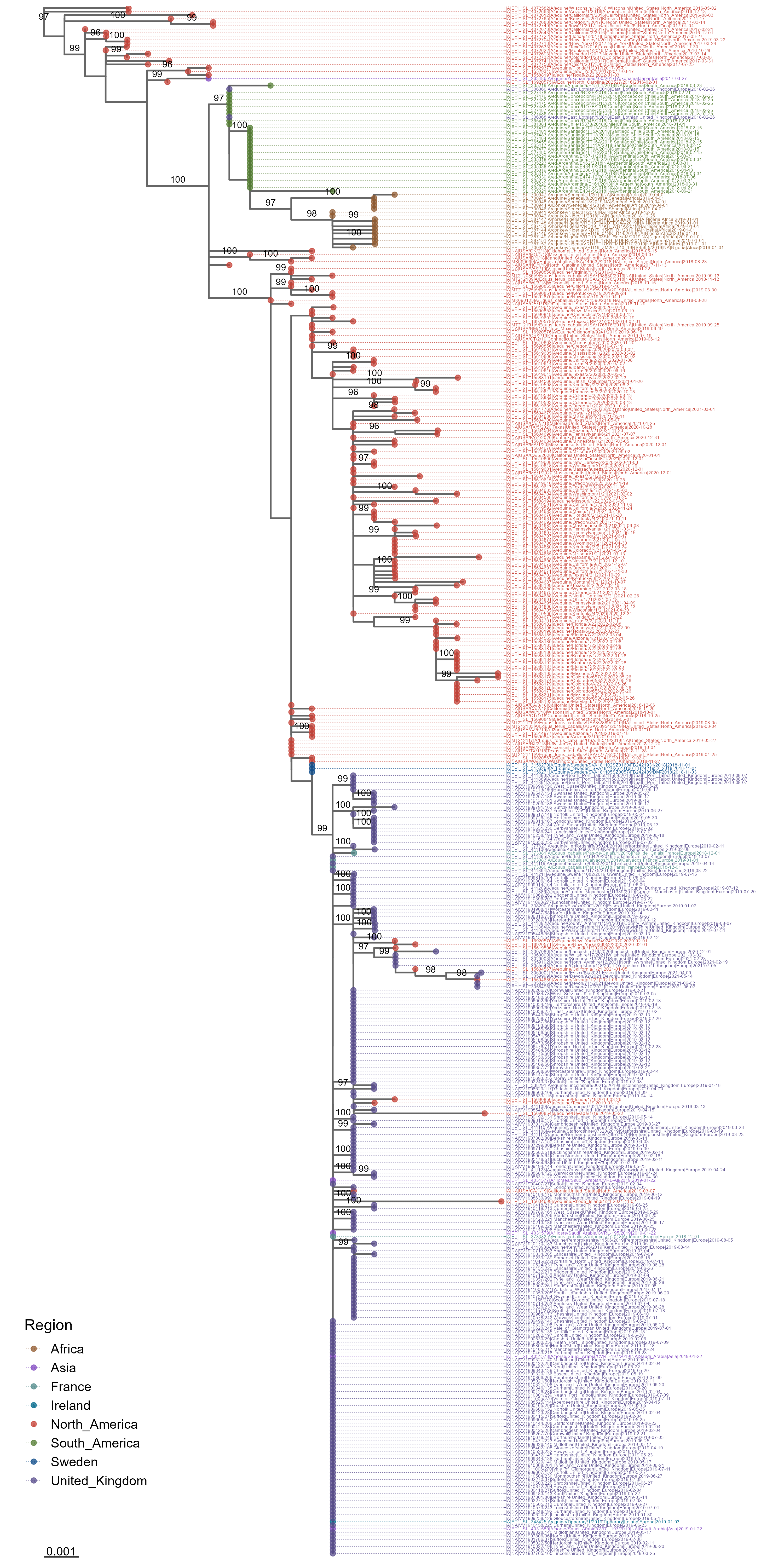

Supplement: S10 Fig — Maximum Likelihood tree obtained from the analysis of the HA-only dataset using IQTree. Values on branches represent ultrafast bootstrap values (>95), obtained from 1000 pseudoreplicates. Tips are coloured according to Location: “Countries” for European isolates and “Region” for other continents. (TIFF) [file ppat.1013227.s010.tiff]

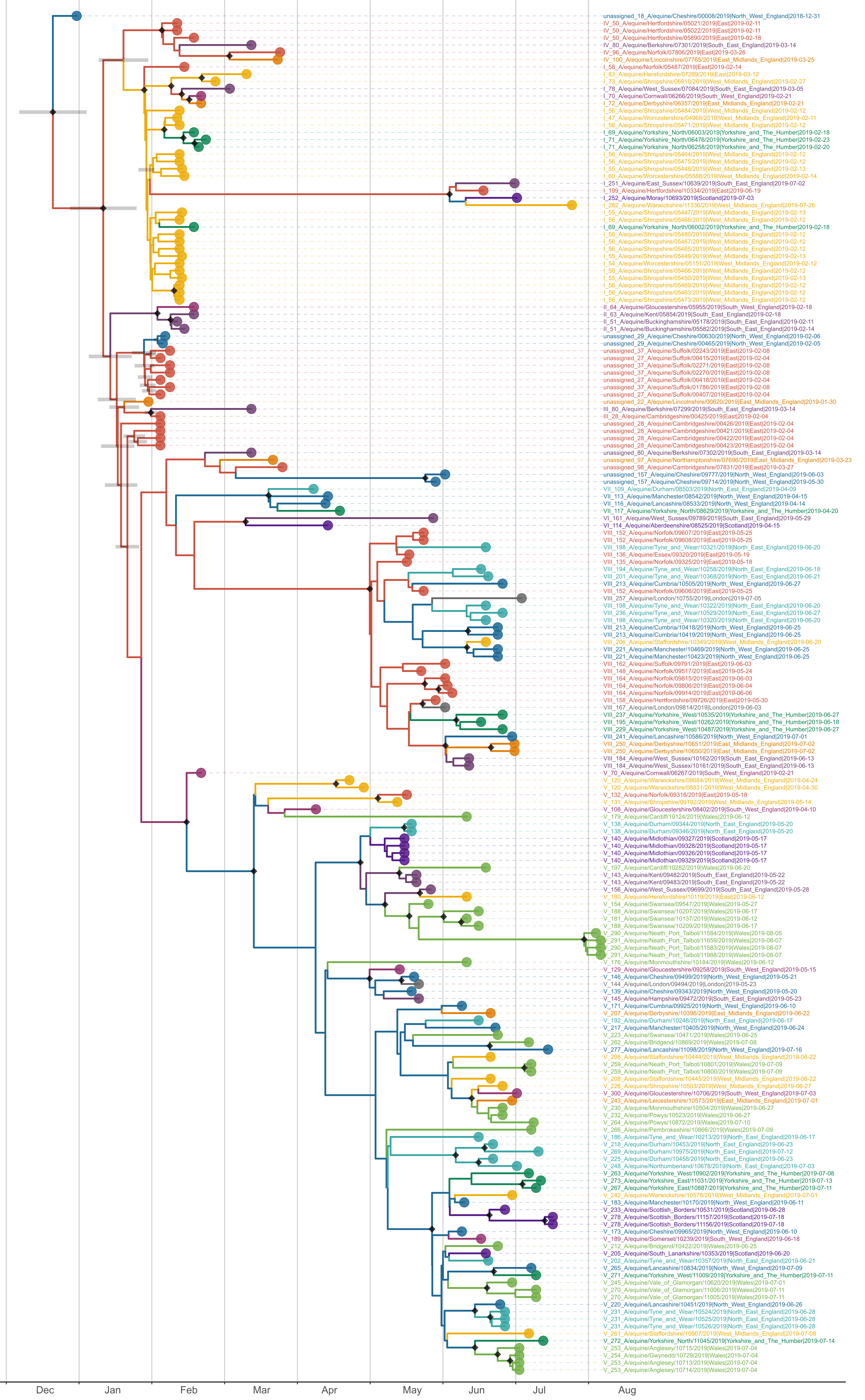

Supplement: S11 Fig — MCC tree obtained from the discrete phylogeographic analysis (shown in Figs 2 and 3A). Colours on tips names and branches correspond to the regions outlined in the lower-left inset. Tips names include the designated viral cluster, outbreak ID, isolate name, ITL 1 region and collection date. Nodes with posterior probability ≥ 0.9 are labelled with diamonds. (TIFF) [file ppat.1013227.s011.tiff]

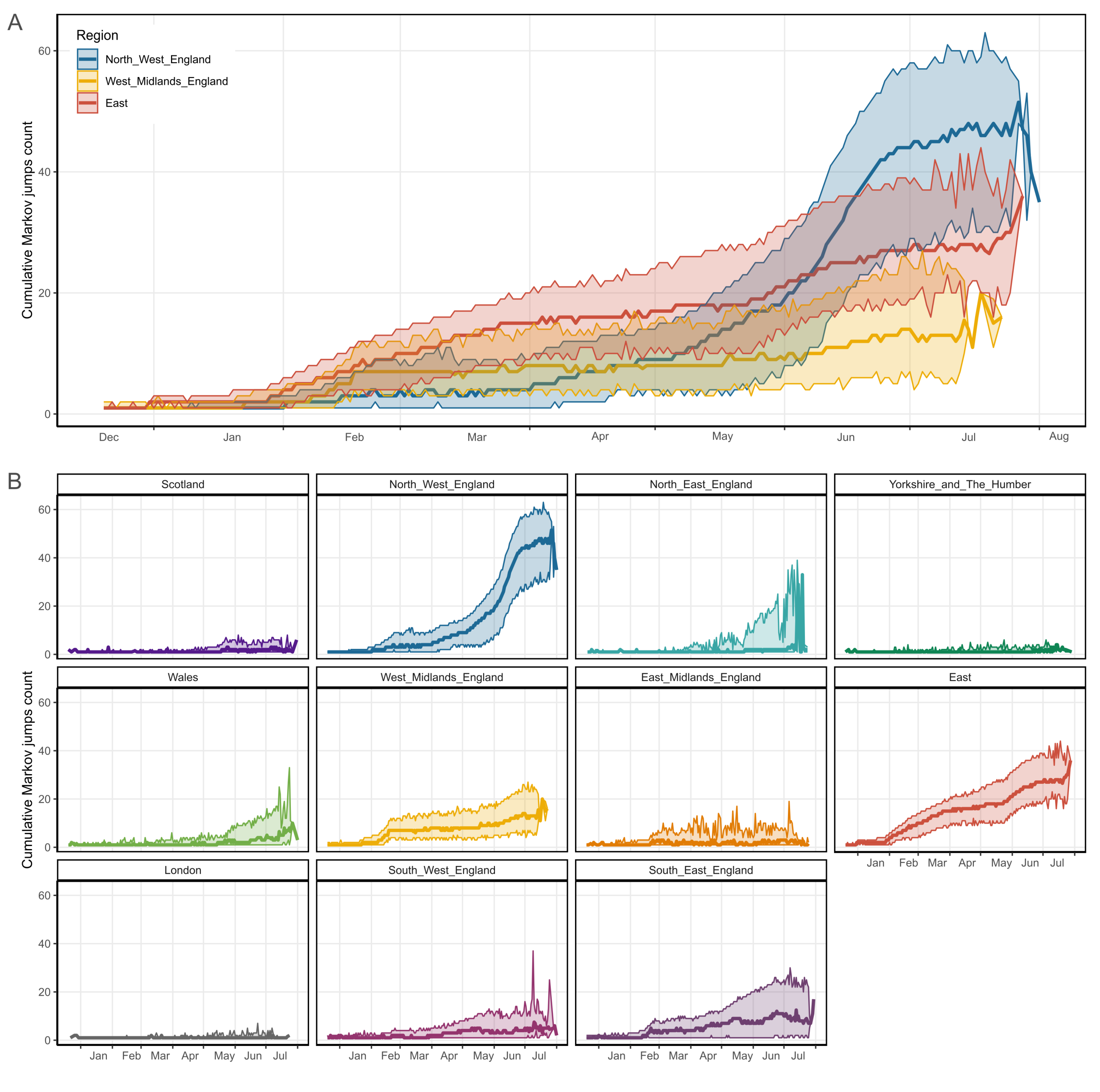

Supplement: S12 Fig — The values represent the cumulative Markov jump counts per day from each UK region to (any) other locations, summarized as the median with 95% HPD intervals per tree across the posterior distribution. (A) Cumulative number of transitions for the three main source regions: East, West Midlands, and North West England. (B) Separate panels displaying the cumulative number of transitions for each UK region independently. (TIFF) [file ppat.1013227.s012.tiff]

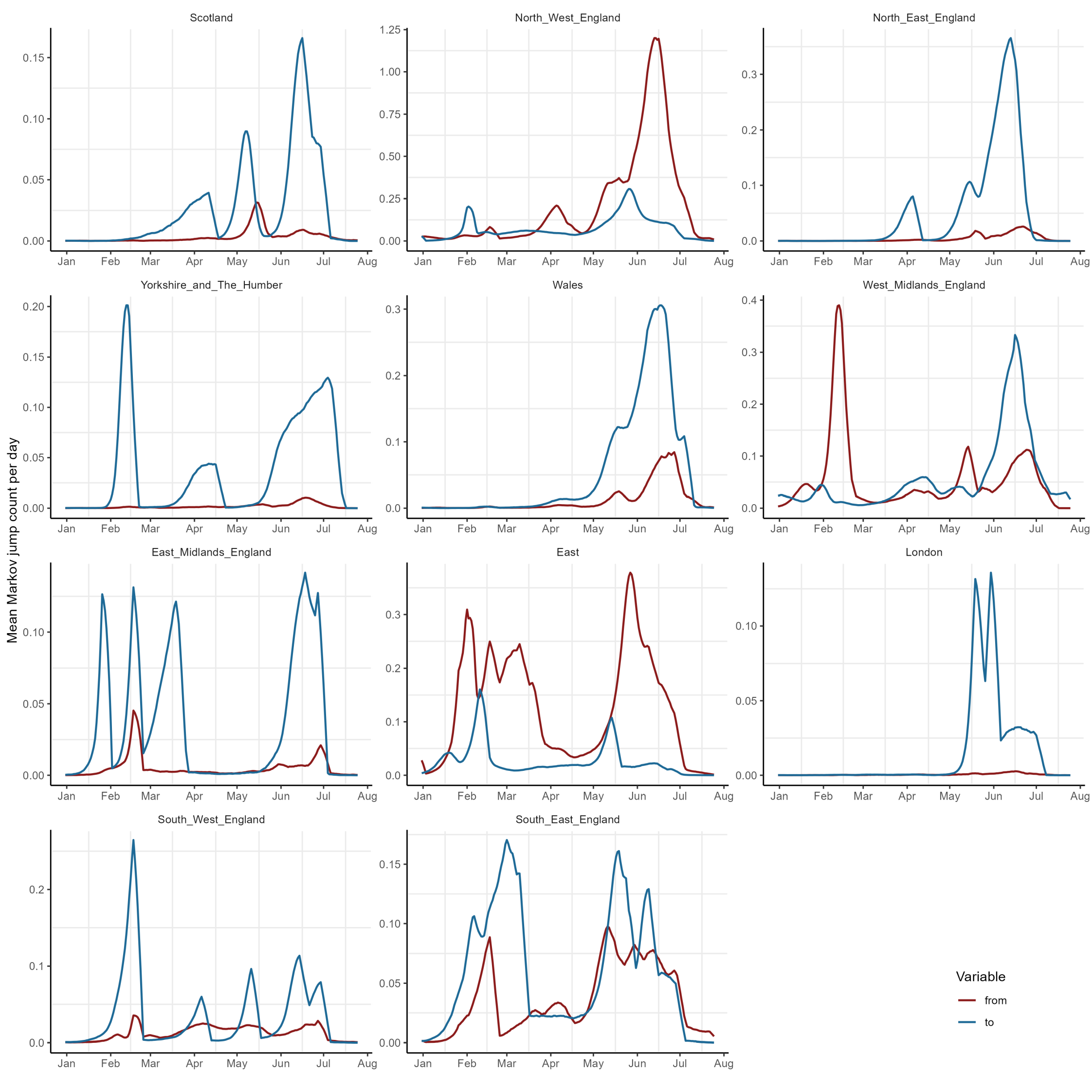

Supplement: S13 Fig — The values represent the mean of the Markov jump count per day from (in red) and to (in blue) each region. The y-axis between the facets is not drawn to scale to enhance the visualization of the curves in regions with low jump counts. The values in the y-axis was calculated as mean number of the Markov jump per day across all trees in the posterior distribution and smoothed using a 7-day centred rolling mean. (TIF) [file ppat.1013227.s013.tif]

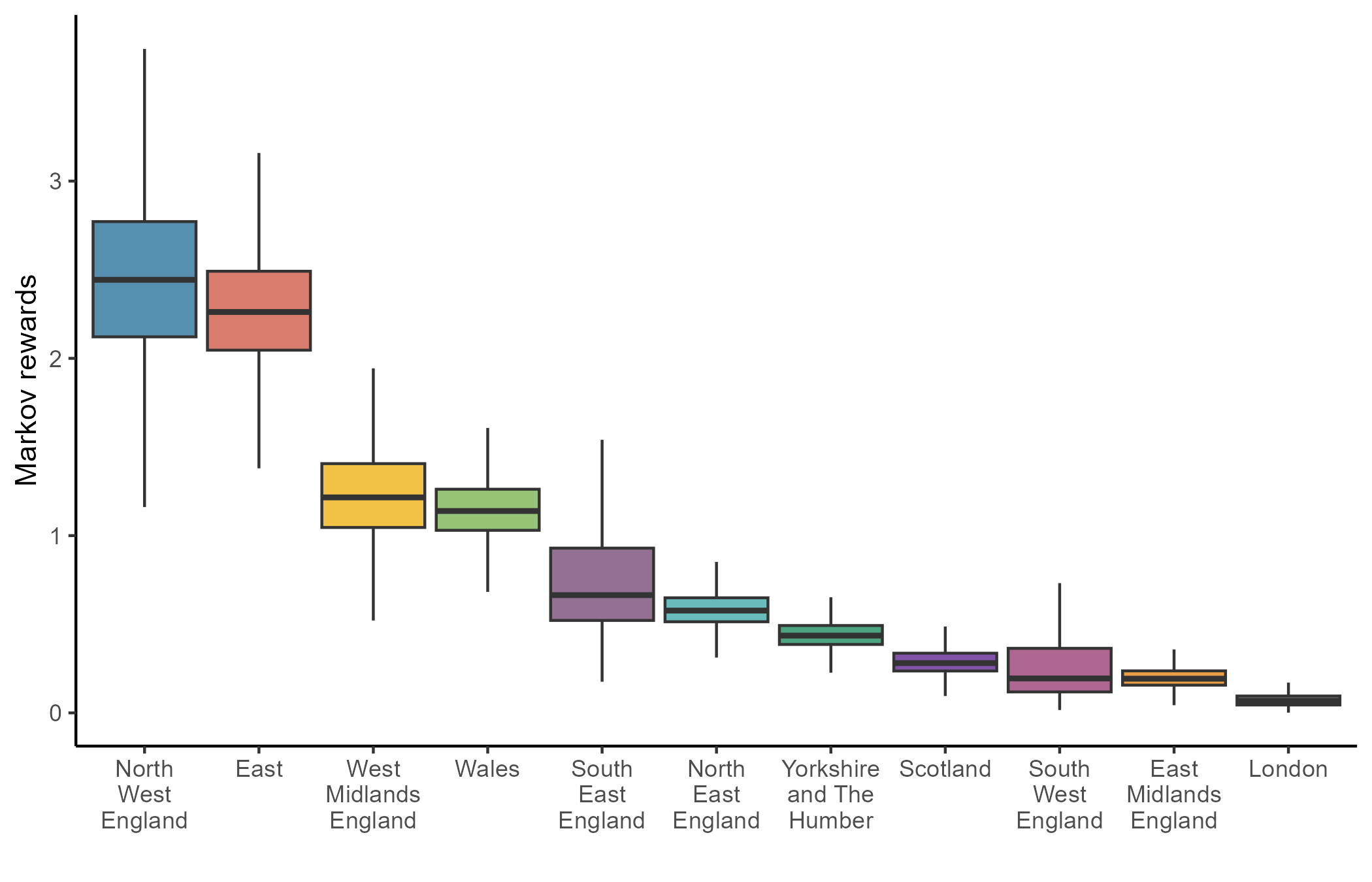

Supplement: S14 Fig — The boxplot of each region depicts the density distribution of the total time spent (boxes show the median and HPD80 interval). (TIFF) [file ppat.1013227.s014.tiff]

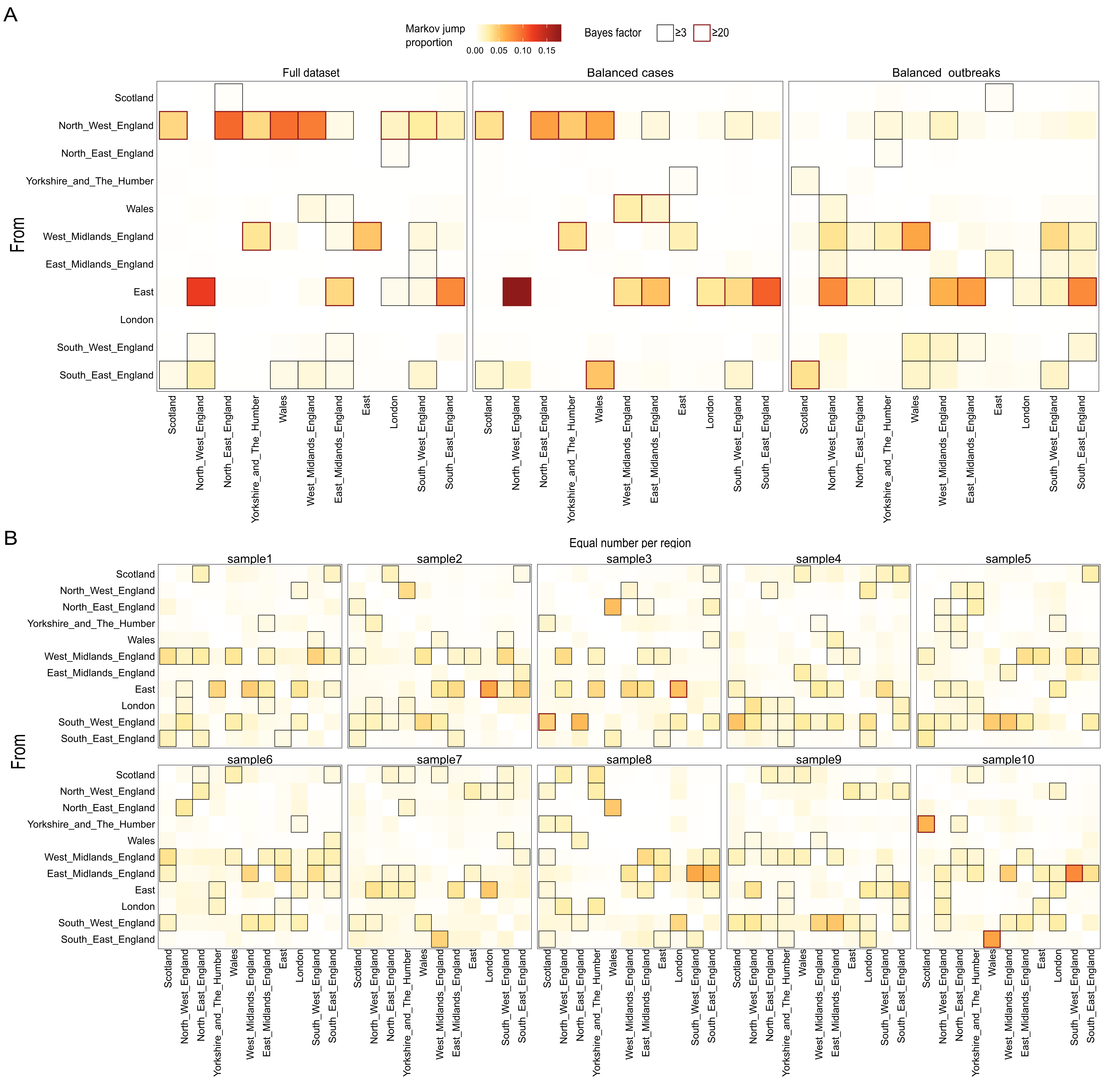

Supplement: S15 Fig — (A) Results from the full dataset are compared to datasets down sampled by the number of confirmed cases and by the number of outbreaks or affected premises per region. (B) Results from replicates (n = 10) of datasets down sampled to an equal number of sequences per region. The heatmap colours represent the proportions of between-region Markov jumps relative to the total number of jumps in each analysis. Links supported by Bayes factors (BF) are highlighted by black (≥3) or red (≥20) edges. (TIF) [file ppat.1013227.s015.tif]

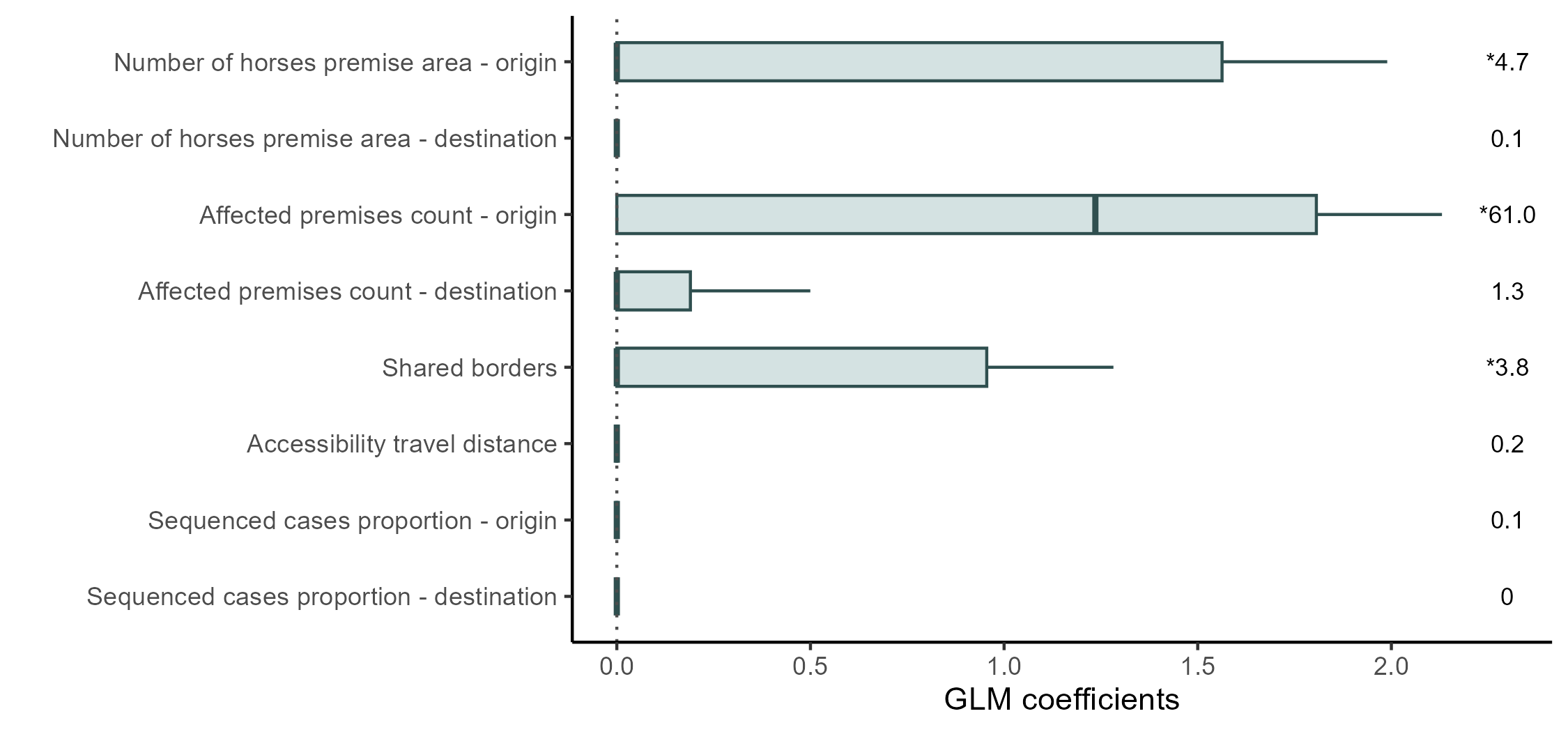

Supplement: S16 Fig — The boxplot represents the contributions of each predictor when included in the model (boxes show the median and HPD80 interval). The BF associated with each predictor considered in the GLM are reported on the right, with supported ones (BF ≥ 3) labelled with an asterisk. (PNG) [file ppat.1013227.s016.png]

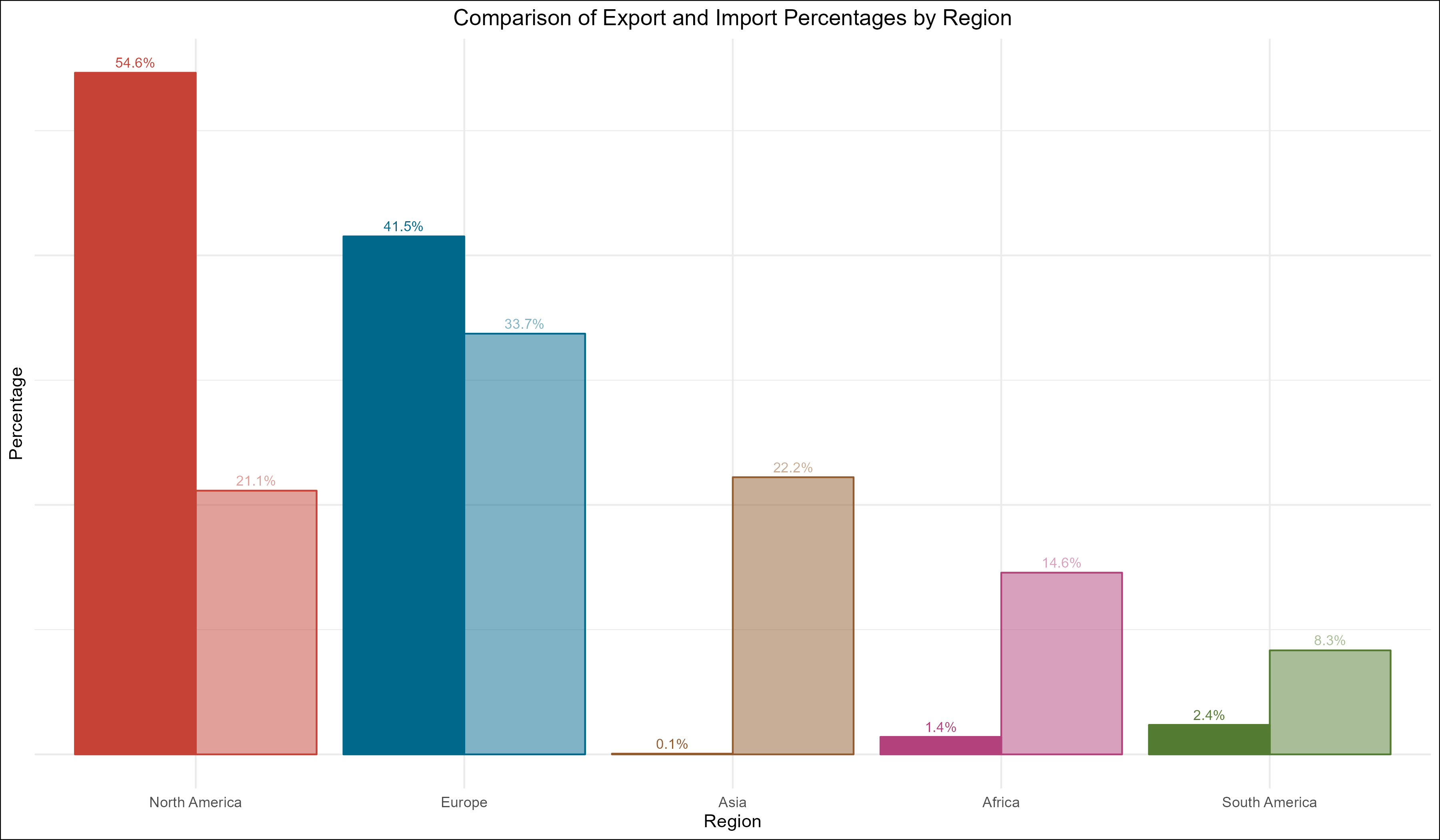

Supplement: S17 Fig — Markov jumps counts obtained from the phylodynamic analysis in BEAST. The bars represent the percentage of the total Markov Jumps count from (solid-coloured bars) and to (transparent bars) each region. (TIFF) [file ppat.1013227.s017.tiff]

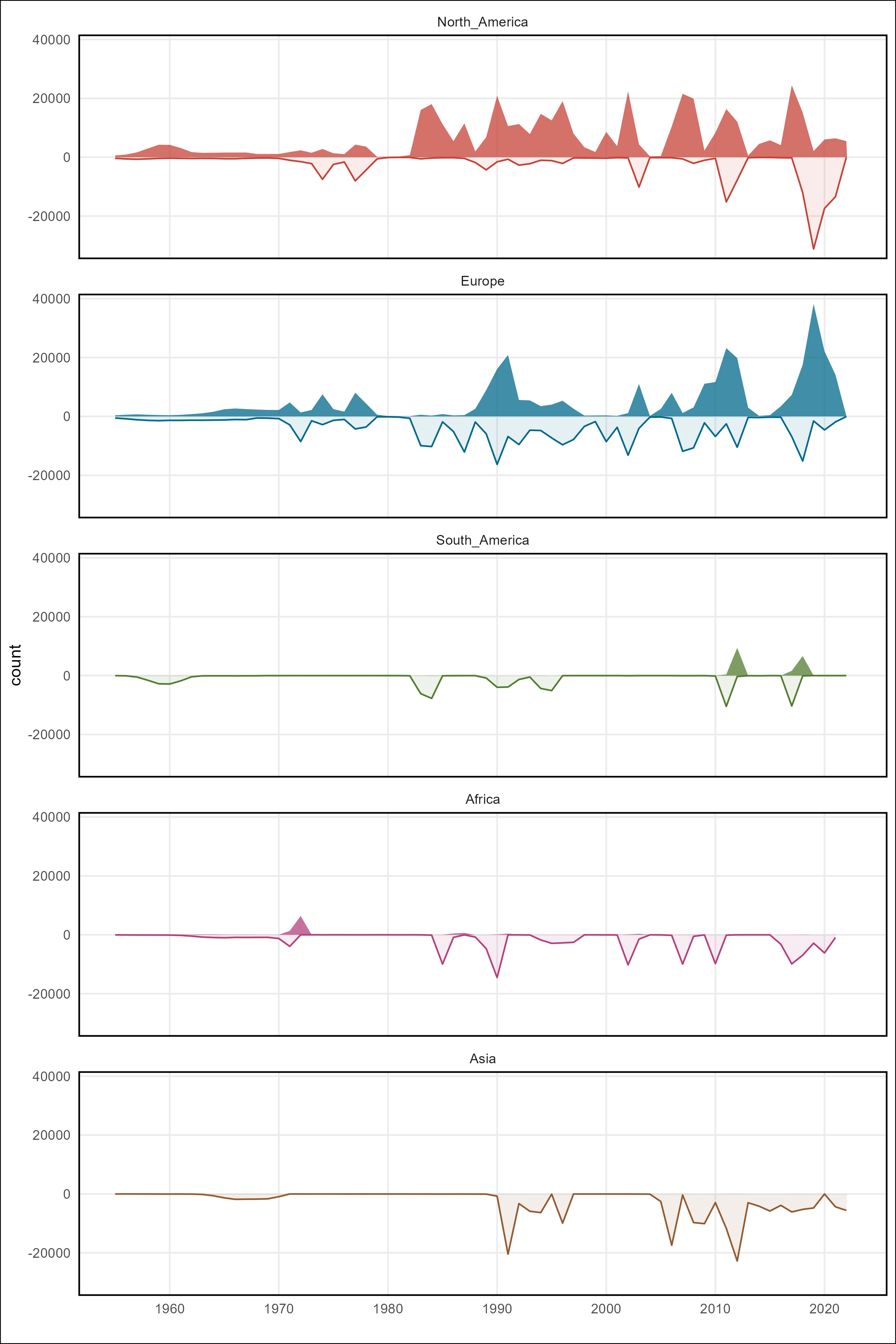

Supplement: S18 Fig — Markov jumps counts obtained from the phylodynamic analysis in BEAST. The values represent the median of the Markov jump count per year “from” (positive y-axis) and “to” (negative y-axis) each region. (TIFF) [file ppat.1013227.s018.tiff]

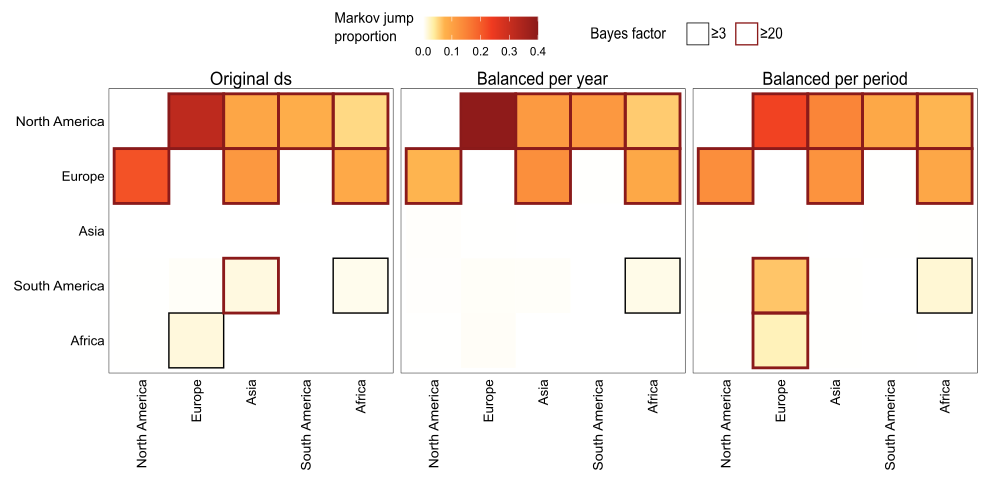

Supplement: S19 Fig — Comparison of proportions of between-region Markov jumps counts and rewards in the (i) original dataset, (B) the balanced number of sequences per region per year and, (C) balanced number of sequences per region per phylogenetic period. (TIFF) [file ppat.1013227.s019.tiff]

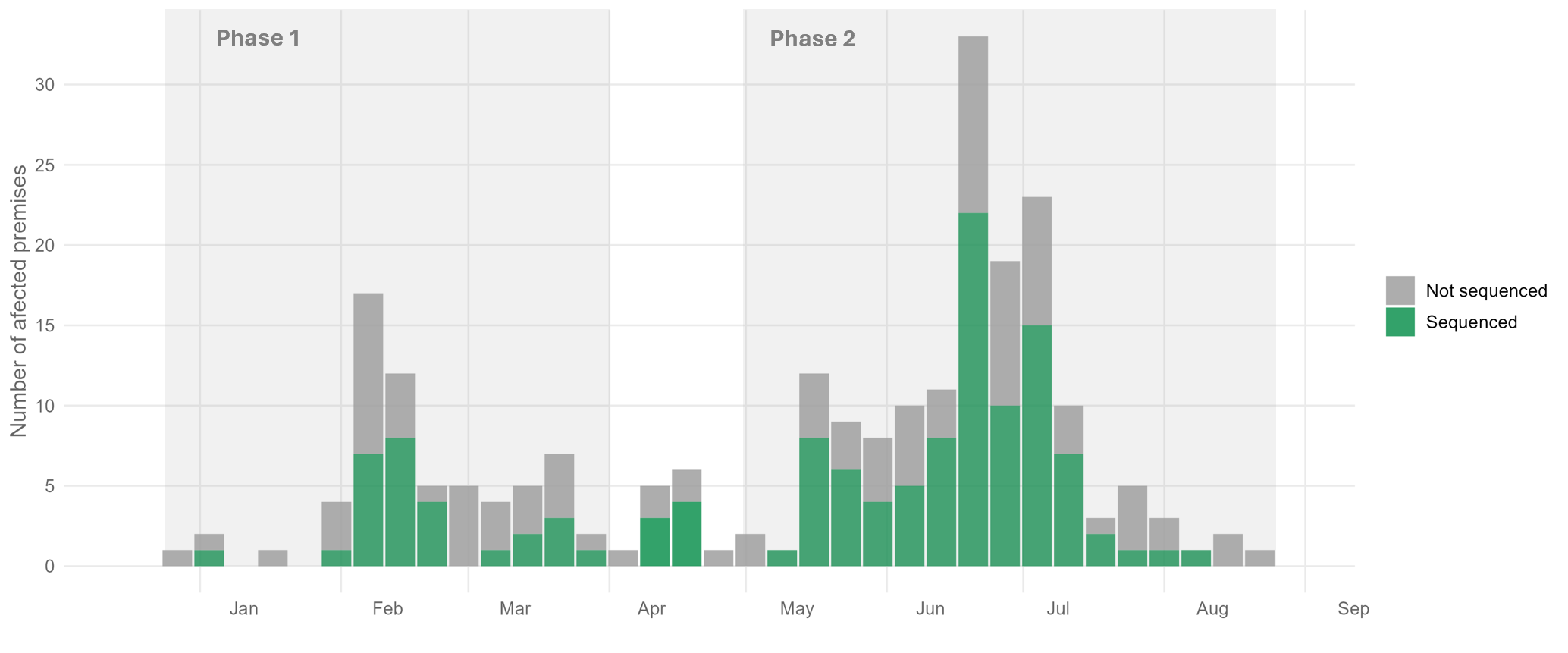

Supplement: S20 Fig — Epizootic curve of affected premises reported per epidemiological week and the corresponding sequencing status. Shaded areas indicate the first and second epizootic phases as defined in [35]. (TIFF) [file ppat.1013227.s020.tiff]

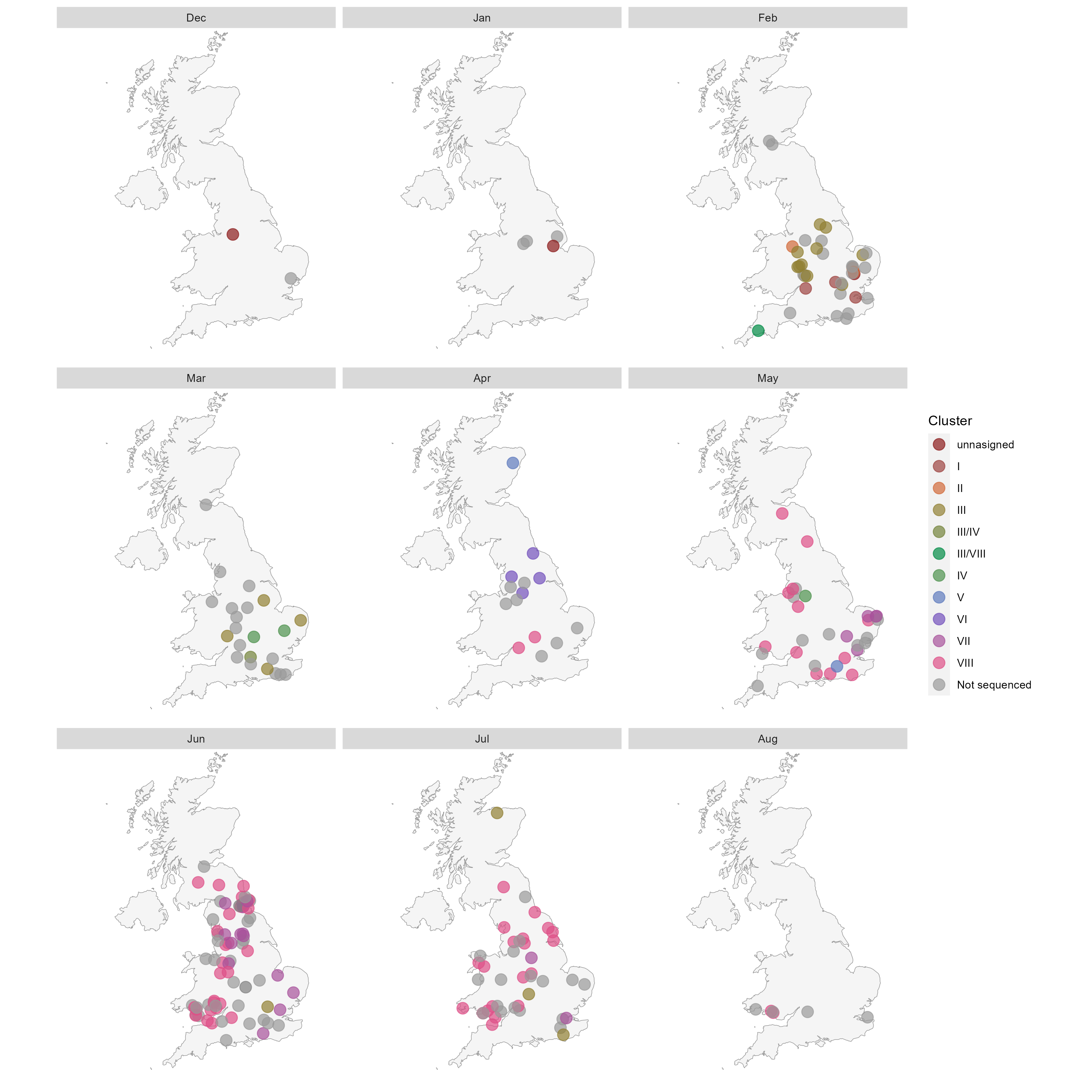

Supplement: S21 Fig — Each circle corresponds to a lab-confirmed facility coloured according to the viral cluster identified. Base UK map shapefile sourced from Natural Earth (https://www.naturalearthdata.com/) public domain. (TIFF) [file ppat.1013227.s021.tiff]
