# Supplementary material for: Multiple introductions of equine influenza virus into the United Kingdom resulted in widespread outbreaks and lineage replacement
Source: PLoS Pathog. 2025 Jun 9;21(6):e1013227. doi: 10.1371/journal.ppat.1013227 (PMC12236680; doi:10.1371/journal.ppat.1013227)
Supplement: S2 Table — AI: association index; PS: parsimony score; MC: Monophyletic Clade size statistic; CI: confidence interval. (DOCX) [file ppat.1013227.s023.docx]

**S2 Table**

| Statistic | observed mean | lower 95% CI | upper 95% CI | null mean | lower 95% CI | upper 95% CI | significance level |
| --- | --- | --- | --- | --- | --- | --- | --- |
| AI | 10.368 | 9.553 | 11.179 | 11.699 | 10.906 | 12.332 | 0.000 |
| PS | 70.376 | 68.000 | 72.000 | 80.245 | 76.897 | 83.173 | 0.000 |
| MC Scotland | 1 | 1.000 | 1.000 | 1.071 | 1.000 | 1.321 | 1.000 |
| MC North West England | 1.103 | 1.000 | 2.000 | 1.536 | 1.001 | 2.091 | 1.000 |
| MC North East England | 2.04 | 2.000 | 2.000 | 1.217 | 1.000 | 2.000 | 0.090 |
| MC Yorkshire and The Humber | 1 | 1.000 | 1.000 | 1.096 | 1.000 | 2.000 | 1.000 |
| MC Wales | 2.672 | 2.000 | 3.000 | 1.658 | 1.075 | 2.519 | 0.002* |
| MC West Midlands England | 1.691 | 1.000 | 3.000 | 1.298 | 1.000 | 2.000 | 0.090 |
| MC East Midlands England | 1 | 1.000 | 1.000 | 1.069 | 1.000 | 1.476 | 1.000 |
| MC East | 1.712 | 1.000 | 2.000 | 1.719 | 1.083 | 2.506 | 0.360 |
| MC London | 1 | 1.000 | 1.000 | 1.024 | 1.000 | 1.053 | 1.000 |
| MC South West England | 1 | 1.000 | 1.000 | 1.079 | 1.000 | 1.487 | 1.000 |
| MC South East England | 1.999 | 2.000 | 2.000 | 1.347 | 1.000 | 2.059 | 0.120 |

**Summary of association index from BaTS analysis.** AI: association index; PS: parsimony score; MC: Monophyletic Clade size statistic; CI: confidence interval.
