# Supplementary material for: Multiple introductions of equine influenza virus into the United Kingdom resulted in widespread outbreaks and lineage replacement
Source: PLoS Pathog. 2025 Jun 9;21(6):e1013227. doi: 10.1371/journal.ppat.1013227 (PMC12236680; doi:10.1371/journal.ppat.1013227)
Supplement: S3 Table — The BF were obtained using a model averaging procedure (BSSVS) in BEAST and only transitions with positive or strong support (BF ≥ 3) are shown, sorted from high to low. (DOCX) [file ppat.1013227.s024.docx]

**S3 Table**

| **From** | **To** | **BF full DS** | **BF Hierarchical** |
| --- | --- | --- | --- |
| North West England | North East England | **2872.87** | **81.97** |
| East | North West England | **1257.12** | **7.62** |
| West Midlands England | Yorkshire and The Humber | **1219.87** | **24.56** |
| East | South East England | **449.96** | **49.58** |
| North West England | West Midlands England | **408.63** | **81.12** |
| North West England | Wales | **368.92** | **17.51** |
| West Midlands England | East | **189.72** | **191.98** |
| North West England | Scotland | **117.74** | **7.63** |
| East | East Midlands England | **91.90** | **4.47** |
| North West England | Yorkshire and The Humber | **49.53** | **22.23** |
| North West England | London | **24.94** | **5.58** |
| North West England | South West England | **22.30** | 2.48 |
| North West England | South East England | **10.26** | 1.63 |
| South East England | South West England | **9.09** | **8.57** |
| South East England | West Midlands England | **6.86** | **3.23** |
| South East England | North West England | **5.84** | **34.30** |
| East | London | **5.32** | **3.62** |
| South East England | Wales | **5.04** | 1.14 |
| Wales | East Midlands England | **5.00** | **6.91** |
| South East England | Scotland | **4.82** | **3.04** |
| East Midlands England | South West England | **4.56** | **6.45** |
| North West England | East Midlands England | **4.53** | 1.91 |
| Wales | West Midlands England | **4.25** | **3.42** |
| West Midlands England | South West England | **4.21** | 2.89 |
| South West England | East Midlands England | **3.96** | 2.44 |
| East | South West England | **3.81** | 0.24 |
| West Midlands England | East Midlands England | **3.62** | 2.41 |
| South West England | North West England | **3.61** | 2.35 |
| Scotland | North East England | **3.16** | **3.08** |
| North East England | London | **3.13** | **3.95** |
| South East England | East Midlands England | **3.06** | **16.41** |
| South West England | South East England | 2.81 | **10.80** |
| West Midlands England | South East England | 2.77 | **3.77** |
| South West England | West Midlands England | 2.69 | **6.20** |
| Wales | South West England | 2.06 | **3.31** |
| Yorkshire and The Humber | Scotland | 1.50 | **3.55** |
| North East England | North West England | 1.37 | **5.21** |
| East | North East England | 0.99 | **6.12** |

**Overview of the well-supported transitions between the UK regions**. BF obtained for the full dataset (BF full DS) and the hierarchical model (BF Hierarchical) analyses.

The BF were obtained using a model averaging procedure (BSSVS) in BEAST and only transitions with positive or strong support (BF ≥3) in at least one analysis are shown, sorted from high to low according to the BF in the full dataset analysis. Transitions supported in both analyses are shown in bold.
