# Supplementary material for: Multiple introductions of equine influenza virus into the United Kingdom resulted in widespread outbreaks and lineage replacement
Source: PLoS Pathog. 2025 Jun 9;21(6):e1013227. doi: 10.1371/journal.ppat.1013227 (PMC12236680; doi:10.1371/journal.ppat.1013227)
Supplement: S4 Table — Bayes Factor (BF) was calculated with a prior odds of 0.09051 (prior probability distribution = 0.083). Predictors included in the model (BF ≥ 3) are indicated with an asterisk. (DOCX) [file ppat.1013227.s025.docx]

**S4 Table**

| Predictor | Coefficient Mean | Coefficient HPD95% interval | Coeff indicator Mean | BF | Coeff time indicator Mean | Coeff time indicator HPD95% interval |
| --- | --- | --- | --- | --- | --- | --- |
| Number of horses premise area - origin | 0.3827 | [-3.6453, 3.5073] | 0.2974 | 4.68* | 0.4065 | [-0, 1.8152] |
| Number of horses premise area - destination | -0.0030 | [-3.8662, 3.9023] | 0.0094 | 0.11 | 0.0021 | [-0, -0] |
| Affected premises count - origin | 1.1335 | [-1.4231, 2.7528] | 0.8466 | 60.98* | 1.1347 | [-0, 1.9751] |
| Affected premises count - destination | 0.0161 | [-3.7531, 3.7859] | 0.1088 | 1.35 | 0.0423 | [0, 0.3965] |
| Shared borders | 0.2430 | [-3.628, 3.7774] | 0.2557 | 3.80* | 0.2239 | [-0, 1.1454] |
| Accessibility travel distance | 0.0017 | [-3.9443, 3.9408] | 0.0136 | 0.15 | -0.0030 | [-0, -0] |
| Sequenced cases proportion - origin | -0.0256 | [-4.0182, 3.7872] | 0.0128 | 0.14 | -0.0045 | [-0, 0] |
| Sequenced cases proportion - destination | -0.0355 | [-4.1703, 3.6937] | 0.0041 | 0.05 | 0.0005 | [-0, -0] |

**Inclusion support statistics for viral spread predictors evaluated in the GLM analysis.** Bayes Factor (BF) was calculated with a prior odds of 0.09051 (prior probability distribution = 0.083). Predictors included in the model (BF ≥ 3) are indicated with an asterisk .
