# Supplementary material for: Multiple introductions of equine influenza virus into the United Kingdom resulted in widespread outbreaks and lineage replacement
Source: PLoS Pathog. 2025 Jun 9;21(6):e1013227. doi: 10.1371/journal.ppat.1013227 (PMC12236680; doi:10.1371/journal.ppat.1013227)
Supplement: S6 Table — Df: degrees of freedom; t value: t-statistic value; Pr(>|t|): p-value with significance level codes (***: p < 0.001). (DOCX) [file ppat.1013227.s027.docx]

**S6 Table**

| **Fixed Effects** | | | | | | |
| --- | --- | --- | --- | --- | --- | --- |
| **Variable** | **Status** | **Estimate** | **Std. Error** | **df** | **t value** | **Pr(>\|t\|)** |
|  | Intercept | 4.400281 | 0.079023 | 223.016 | 55.684 | < 2e-16 *** |
| Vaccination status | unvaccinated | reference |  |  |  |  |
|  | vaccinated | -0.661902 | 0.183898 | 196.287 | -3.599 | 0.000404 *** |
| day from sign to sample |  | -0.012793 | 0.002864 | 249.825 | -4.467 | 1.2e-05 *** |
| **Random Effects** | | | | | | |
|  | **Name** | **Variance** | **Std.Dev.** |  |  |  |
| outbreakid | Intercept | 0.2524 | 0.5024 |  |  |  |
| Residual |  | 1.0464 | 1.0229 |  |  |  |

**Summary of Results of Linear Mixed-Effects Model.** Df: degrees of freedom; t value: t-statistic value; Pr(>|t|): p-value with significance level codes (***: p < 0.001).
